# Dynamics of the intratumoural microbiome across malignant transformation and treatment in breast cancer

**DOI:** 10.1002/ctm2.70492

**Published:** 2025-10-01

**Authors:** Liuliu Quan, Mengwu Shi, Zixuan Yang, Huiteng Rong, Jingyi Zhou, Die Sang, Jing Xu, Jian Yue, Shuyue Chen, Jingsong Liu, Peng Yuan

**Affiliations:** ^1^ Department of Medical Oncology, National Cancer Center, National Clinical Research Center for Cancer, Cancer Hospital Chinese Academy of Medical Sciences and Peking Union Medical College Beijing China; ^2^ Department of VIP Medical Oncology, National Cancer Center, National Clinical Research Center for Cancer, Cancer Hospital Chinese Academy of Medical Sciences and Peking Union Medical College Beijing China; ^3^ Department of Data Science, School of Data Science The Chinese University of Hong Kong Shenzhen China; ^4^ The Second Clinical College Chongqing Medical University Chongqing China; ^5^ Department of Medical Oncology Beijing Chaoyang District Sanhuan Cancer Hospital Beijing China; ^6^ Department of Mathematics, College of Science Zhejiang Sci‐Tech University Hangzhou Zhejiang China

**Keywords:** 16s RNA, breast cancer, cancer development, intratumoural microbiome, malignant transformation, neoadjuvant therapy, predictive model

## Abstract

Breast cancer (BC) is the most common malignancy in women, yet the dynamics of the intratumoural microbiome during tumour initiation, progression, and treatment remain poorly understood. Prior studies are predominantly cross‐sectional and limited by indirect microbial inference from RNA‐seq data. This study presents a comprehensive analysis of intratumoural microbiota across breast tissue samples by high‐depth 16S rRNA sequencing (11 W tags), featuring two longitudinally paired cohorts for dynamic microbial profiling during tumour progression and treatment. Samples included 165 benign nodules (82 non‐transforming, 83 that later progressed to cancer with matched malignant tissues); 180 primary BC tissues and 165 benign controls; and 101 neoadjuvant therapy (NAT) specimens (15 pCR, 86 non‐pCR, with paired pre/post‐treatment samples). We identified a cluster of taxa (Aeromicrobium, Halomonas, Dietzia, Nesterenkonia, Delftia, Nitriliruptor) depleted in nodules undergoing malignant transformation, declining with disease progression and partially restored after NAT, with transient enrichment early in transformation. Opposing trends were observed for *Paenibacillus* and *Methyloversatilis*. These changes corresponded to shifts in amino acid, lipid, and glycan metabolism. FISH and TEM analyses identified *Paenibacillus pasadenensis* and *Halomonas hamiltonii* within tumour cells, with opposing effects on tumour proliferation and activation. In addition, we developed two predictive models with high clinical relevance: one stratifying malignancy risk in nodules, and another predicting NAT response, both of which achieved strong performance in external validation. This longitudinal characterisations of intratumoural microbiota during breast tumourigenesis and treatment offer novel insights for precision oncology and microbiome‐based interventions in breast cancer.

## Graphical abstract

1. Certain taxa correlated with tumour progression, others inversely; microbial changes tracked malignancy and metabolism.

2. *Paenibacillus pasadenensis* and *Halomonas hamiltonii* differentially regulate breast cancer cell growth and migration in vitro.

3. Two microbiota‐based models were developed: one to predict malignant transformation of benign breast nodules, and another to predict response to neoadjuvant therapy.

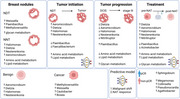

## 1 INTRODUCTION

Breast cancer (BC) remains one of the most prevalent cancers among women worldwide.^1^ In 2022, around 2.3 million new BC cases were identified worldwide, with an estimated 666 000 deaths attributable to the disease. BC accounted for 23.8% of all newly diagnosed cancers and 15.4% of cancer‐related deaths among women, representing a major cause of cancer mortality in this population.^2^ Early detection through screening plays a critical role in improving clinical outcomes. In recent years, the occurrence of breast nodules has been on the rise steadily, particularly among women around the age of 40. With the widespread application of imaging modalities such as magnetic resonance imaging, mammography, and ultrasound, a substantial number of breast nodules are now identified at an early stage.^3^, ^4^ Although traditional imaging modalities and histopathological evaluation can identify existing lesions of breast nodules, they lack prospective predictive power regarding future malignant transformation. Therefore, the early and accurate identification of benign nodules with malignant potential has become a pressing challenge in clinical practice.

In the search for novel risk stratification strategies, increasing attention has been directed toward the intratumoural microbiota in light of its potential involvement in the initiation and evolution of tumours.^1^, ^3^, ^5^ The intratumoural microbiota encompasses microorganisms, including bacteria, viruses, and fungi, that inhabit within tumour tissues and their surrounding microenvironment. This concept initially emerged from studies linking *Helicobacter pylori* infection to gastric cancer and has since been substantiated across multiple tumour types.^6^ The intratumoural microbiota in BC has been closely linked to both the onset and advancement of the disease. For instance, tumour tissues from BC patients frequently exhibit enrichment of *Pseudomonas*, *Proteus*, and *Nitrosomonas* Species, whereas *Sphingomonas* and *Corynebacterium* are notably reduced.^7^ However, most existing studies are cross‐sectional in design and therefore cannot capture the dynamic alterations of the intratumoural microbiota accompanying the transformation of benign breast nodules into malignant tumours. Investigating the temporal evolution of the intratumoural microbiota throughout malignant transformation may offer novel insights into the mechanisms underlying breast carcinogenesis.

In BC, the intratumoural microbiota also shows a close association with clinical characteristics. Studies have shown that the abundance of *Lacibacter* and *Ezakiella* is significantly increased in advanced‐stage BC tissues.^8^ Distinct microbial compositions have also been observed among different molecular subtypes. For example, *Alkanindiges* and *Micrococcus* are relatively depleted in estrogen receptor (ER)‐positive BCs; *Pelomonas* is enriched in progesterone receptor (PR)‐positive cases; and human epidermal growth factor receptor 2 (HER2)‐positive BCs exhibit significant enrichment of *Cloacibacillus*, and various anaerobic bacteria. These microbial distinctions suggest that the intratumoural microbiota may be pivotal in the biological heterogeneity of BC. Previous studies have further demonstrated that intratumoural bacteria can not only disseminate to distant organs through the circulatory system but also collaborate with host tumour cells to establish new microenvironments, thereby promoting cancer progression and metastasis.^7^

There is growing attention the intratumoural microbiota's potential impact in BC treatment. Neoadjuvant therapy (NAT) was selected as the focus of this study due to its unique potential to provide matched tumour specimens before and after treatment, offering a rare opportunity to investigate dynamic microbiota responses within the same tumour environment over the course of therapy. NAT refers to the administration of chemotherapy, targeted therapy, or immunotherapy in the pre‐surgical phase, with the aims of reducing tumour burden, minimising the risk of postoperative recurrence, and evaluating tumour responsiveness to treatment.^9^ Pathological complete response (pCR), characterised by the absence of tumour cells in both the primary tumour site and regional lymph nodes after NAT, has been widely adopted as a surrogate endpoint in clinical trials of neoadjuvant systemic therapies for BC. Effective methods for predicting treatment response remain lacking, and accurately assessing and forecasting NAT outcomes continues to pose a major clinical challenge.^10^ Consequently, there is an urgent need to identify novel biomarkers for the precise prediction of NAT response and the development of personalised therapeutic strategies. Recent studies have indicated that the intratumoural microbiota and its associated products, including DNA, structural proteins, and metabolites, can directly or indirectly modulate the tumour microenvironment, thereby influencing therapeutic outcomes. Moreover, specific intratumoural microorganisms have been shown to reduce the efficacy of chemotherapeutic agents by altering their chemical structure, contribute to immune evasion, and regulate tumour cell proliferation and metastasis.^11^ While these findings provide a theoretical foundation for the role of the microbiota in cancer therapy, no studies to date have systematically explored the involvement and predictive value of the intratumoural microbiota in the realm of NAT for BC.

In summary, this study aims to dynamically monitor changes in the intratumoural microbiota during the malignant transformation of benign breast nodules through long‐term patient follow‐up, and to explore the association between the intratumoural microbiota and nodule malignancy. Additionally, the study will analyse its associations with tumour characteristics, including tumour subtype, stage, and molecular characteristics, and further evaluate the intratumoural microbiota as a potential biomarker for predicting response to NAT in BC. The ultimate goal of this research is to elucidate the mechanisms by which the intratumoural microbiota influences the initiation, development, and treatment response of BC, thereby providing novel theoretical foundations and strategies for the early diagnosis and precision therapy of the disease.

## 2 METHODS AND MATERIALS

### 2.1 Study population and sample grouping

#### 2.1.1 Patients with breast nodules

The criteria for inclusion in the nodule group were defined as follows: evidence of breast nodules on imaging examinations with a BI‐RADS score of ≥2; histopathological confirmation of benign lesions; age between 18 and 75 years. Exclusion criteria included a history of malignancies; concurrent severe infectious diseases; and incomplete clinical data.

**FIGURE 1:**
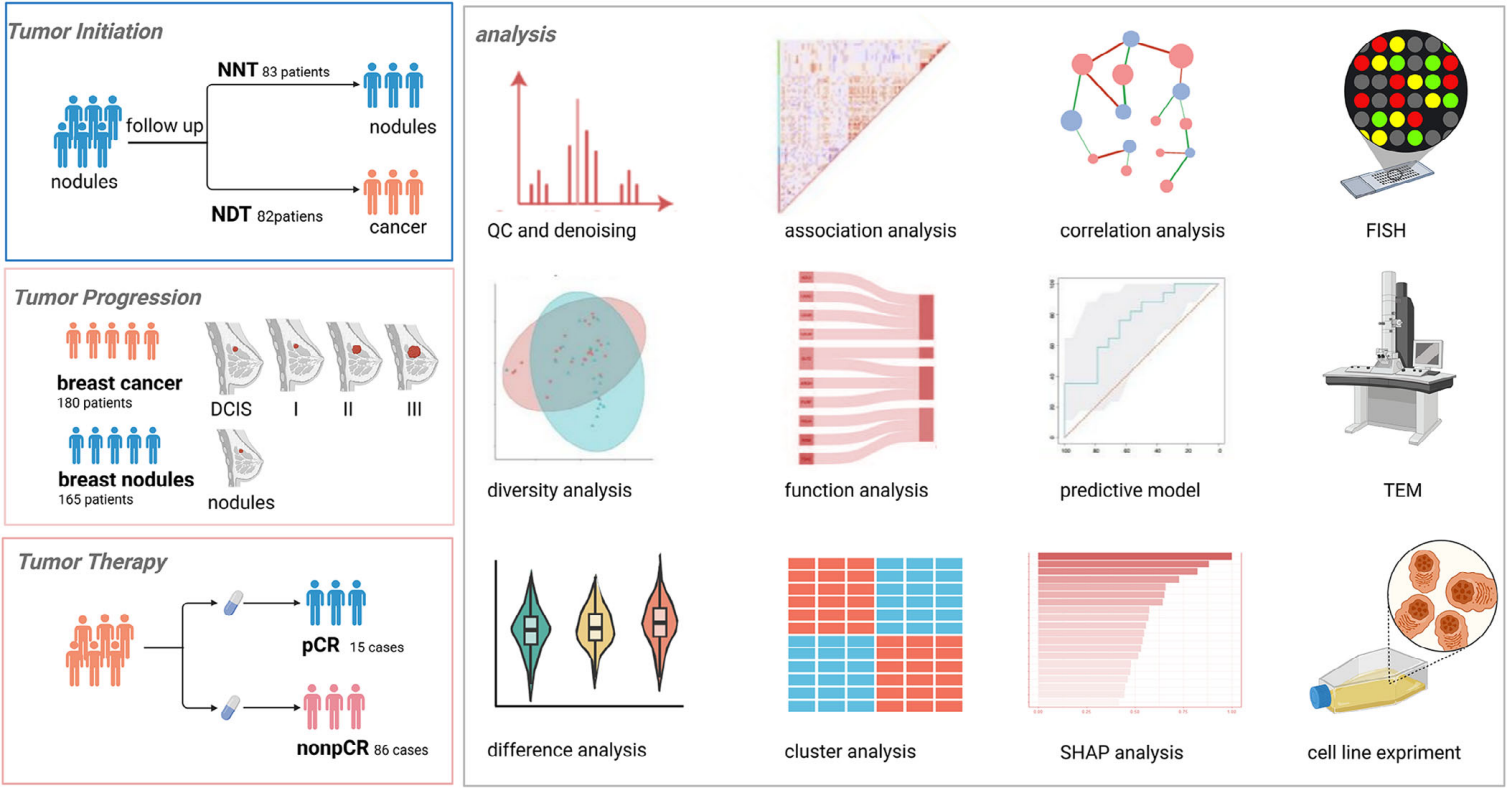
Overview of study design. FISH, fluorescence in situ hybridisation; NDT, neoplastic development group; NNT, non‐neoplastic nodule group; non‐pCR, non‐pathological complete response; pCR, pathological complete response; QC, quality control; SHAP, SHapley Additive exPlanations; TME, transmission electron microscopy.

Based on pathological changes observed during follow‐up, all enrolled patients were further classified into two groups: the non‐neoplastic nodule group (NNT, *n* = 82), comprising patients whose nodules consistently exhibited benign histopathology, and the neoplastic development group (NDT, *n* = 83), consisting of patients whose nodules progressed to malignant breast tumours. Microbiome sequencing analysis was performed on nodule tissue samples collected at baseline for all patients. For patients in the NDT group, the malignant tumour tissues confirmed during follow‐up were also subjected to microbiome sequencing (Figure 1).

#### 2.1.2 Patients undergoing neoadjuvant therapy

Patients undergoing NAT were enrolled according to the following criteria: a histopathological diagnosis of BC (stage I–III) and receipt of neoadjuvant chemotherapy. Exclusion criteria included prior use of antibiotics, antitumour agents, or hormonal therapies before NAT; a history of other malignancies; and the presence of severe infectious diseases or systemic dysfunctions such as hepatic or renal insufficiency. All enrolled patients received NAT regimens formulated by their attending physicians in accordance with established clinical guidelines. Based on postoperative pathological evaluation, patients were classified into two groups according to whether they achieved pCR: the pCR group (patients achieving pCR, *n* = 15) and the non‐pCR group (patients not achieving pCR, *n* = 86). In addition, tumour response to NAT was categorised based on the Miller–Payne (MP) scoring system, ranging from MP grade 1 to grade 5, to further quantify the extent of histological response. To investigate the dynamic changes in the breast tumour microbiota before and after NAT, tissue samples were collected at two time points – preoperative NAT (pre‐NAT) and postoperative NAT (post‐NAT) – for microbiome sequencing analysis, aiming to characterise microbiota evolution during treatment and its potential association with therapeutic response (Figure 1).

#### 2.1.3 Benign and malignant patients

To systematically evaluate the potential role of the intratumoural microbiota in the development of BC, tissue samples from different patient sources were reorganised to establish benign and malignant groups for comparative analysis. The benign group included all tissue samples histopathologically confirmed as benign, comprising samples from patients in NNT group and NDT group. The malignant group included primary tumour tissue samples collected before neoadjuvant therapy from NAT patients, as well as malignant tumour tissues from NDT patients whose lesions progressed to BC during follow‐up (Figure 1).

### 2.2 Sample processing and 16S rRNA gene amplification and sequencing

Tumour samples were retrieved from formalin‐fixed paraffin‐embedded blocks derived from the aforementioned patients. For FFPE samples, only the central tissue portion was used, with the peripheral edges removed to minimise potential contamination. All experiments were conducted under strict aseptic conditions, decontamination of work surfaces and instruments with bleach/70% ethanol and UV irradiation, and the use of sterile, single‐use consumables with filter tips. Negative controls included (1) paraffin/FFPE blank sections and (2) equipment and environmental controls. Sequencing of all negative controls via 16S rRNA revealed only background‐level reads, with no detectable microbial signals.

Total DNA was extracted using a commercial extraction kit (TIANGEN Biotech Co., Ltd., Beijing, China). Briefly, samples were thoroughly mixed with lysis buffer and grinding beads, and then incubated at 70°C for cell lysis. After high‐speed centrifugation (OSE‐MC8, TIANGEN Biotech Co., Ltd.), the supernatant was collected. According to the manufacturer's instructions, binding solution and magnetic beads (DynaMag‐96 Side Skirted, Thermo Fisher Scientific, Waltham, MA, USA) were sequentially added to enrich the DNA, followed by two rounds of anhydrous ethanol washing. After air‐drying the magnetic beads at room temperature, DNA was eluted using elution buffer at 56°C. DNA concentration was quantified using a microplate reader (Synergy HTX, Gene Company Limited) with the 1× dsDNA HS Working Solution (YEASEN Biotechnology, Shanghai, China). Based on DNA concentration and the targeted amplification region, PCR amplification was performed using an automated nucleic acid amplification platform (Bio‐Rad C1000 Thermal Cycler, Bio‐Rad Biotech Co., Ltd.). The target region was the V3–V4 hypervariable region of the 16S rRNA gene, amplified using specific primers 338F (5′‐ACTCCTACGGGAGGCAGCA‐3′) and 806R (5′‐GGACTACHVGGGTWTCTAAT‐3′). The size and integrity of the PCR products were assessed by 1.8% agarose gel electrophoresis (Beijing Bomei Fuxin Technology Co., Ltd.). Sequencing libraries were constructed and subjected to paired‐end sequencing on the Illumina NovaSeq 6000 platform, with an increased sequencing depth of 110 000 tags per sample, providing improved resolution compared to the commonly used 50 000‐tag depth.

### 2.3 Quality control and taxonomic annotation

Raw paired‐end sequencing data generated from the Illumina NovaSeq platform were initially merged using FLASH software(v1.2.11^12^ to combine paired‐end reads. Quality filtering was applied to the merged sequences by Trimmomatic (v0.33)^13^ to eliminate poor‐quality reads and adapter contamination. Subsequently, denoising was carried out by the DADA2 algorithm implemented in the QIIME2 platform (version 2020.6) to generate high‐resolution amplicon sequence variants (ASVs) and eliminate chimeric sequences.^14^ Taxonomic annotation of the obtained ASV sequences was conducted using the RDP Classifier (v2.2)^15^, ^16^ using a confidence threshold of .8, referencing the Silva rRNA gene repository (http://www.arb‐silva.de).^17^

### 2.4 Microbial diversity and differential analysis

Diversity metrics were analysed using the obtained ASV tables using QIIME2. α diversity, was evaluated using the Chao1 and ACE richness indices, as well as the Shannon‐Wiener and Simpson diversity indices. Student's *t*‐test was used to assess α‐diversity differences between groups. β diversity was assessed based on Binary Jaccard distances to evaluate differences in microbial community composition between samples. Principal coordinates analysis (PCoA) was performed using the ‘vegan’ package,^18^ and group differences were quantitatively assessed using Analysis of Similarities (ANOSIM). In addition, differential microbial taxa were identified using Linear Discriminant Analysis Effect Size (LEfSe, http://huttenhower.sph.harvard.edu/lefse/), with a Linear Discriminant Analysis (LDA) score threshold set at 4.0.^19^ For comparisons involving more than two groups, LEfSe identified taxa with statistically significant differences across all groups. The Wilcoxon rank‐sum test was applied for pairwise comparisons, and ANOVA was employed for multiple group comparisons. A paired‐sample *t*‐test was performed to analyse differences between paired samples. *p* Values were adjusted using the Benjamini–Hochberg (BH) correction method.

### 2.5 Co‐occurrence network construction and topological analysis

Spearman correlation coefficients were calculated at the genus level based on the ASV abundance data. A correlation threshold of |*r*| > .4 was applied to define co‐occurrence relationships and construct microbial co‐occurrence networks. In these networks, nodes represent genera, and edges indicate statistically significant positive or negative correlations. Network visualisation and topological analyses – including the number of vertices and edges, the ratio of positive to negative edges, edge density, modularity, clustering coefficient, ​centralisation degree​, centralisation betweenness, centralisation closeness and etc.​ – were performed using the ‘*ggClusterNet* package’ in R.^20^

### 2.6 Functional prediction analysis

The function of the microbiome was predicted using Phylogenetic Investigation of Communities by Reconstruction of Unobserved States 2 (PICRUSt2). This method infers the gene content and functional profiles of microbial communities by aligning ASV sequences from samples to reference databases based on phylogenetic placement. Functional annotation was conducted by referencing multiple databases, including Kyoto Encyclopedia of Genes and Genomes (KEGG, https://www.kegg.jp/) and Clusters of Orthologous Groups (COG, https://www.ncbi.nlm.nih.gov/COG/), to generate predicted functional gene profiles for each sample. *p* Values were adjusted using the BH correction method.

### 2.7 Prediction model construction

For different types of input variables, the following feature selection procedure was applied. For microbial and metabolic pathway variables, values were first transformed into relative abundances. Variables with more than 80% zeros or missing values were excluded. Outlier values were handled by clip processing. Intergroup differential analysis was then performed, and variables showing significant differences were selected. Among the significant variables, the top 20% based on abundance were included as model inputs for both microbial and metabolic pathway features. All clinical features were included in the models.

Diagnostic models were constructed using genus‐level microbial relative abundances, metabolic pathway abundances, and clinical features as input variables. The classification labels included (1) the malignant transformation status of breast nodules and (2) the response to neoadjuvant therapy. Multiple classification algorithms were trained, including Random Forest (RF), Support Vector Machine (SVM), and Logistic Regression (LR). Model performance was evaluated using the area under the receiver operating characteristic curve (AUC). To further validate model stability and generalisability, five‐fold cross‐validation was performed during model training, and the models were also evaluated using an external validation cohort.

### 2.8 External validation cohort

To further validate our findings, we retrospectively collected an independent cohort from Beijing Chaoyang District Sanhuan Cancer Hospital. This cohort comprised 20 patients with breast nodules who underwent pathological evaluation and follow‐up to confirm the nature of the lesions (benign vs. malignant transformation). In addition, we included 20 patients who received neoadjuvant chemotherapy and had corresponding specimens available. These samples were subjected to the same sequencing, preprocessing, and analytical pipeline as described above, and the trained models were applied to evaluate predictive performance in this external dataset.

### 2.9 SHAP value calculation and feature importance assessment

SHapley Additive exPlanations (SHAP) is a model interpretation method based on cooperative game theory that quantifies the marginal contribution of each feature to the model's output.^21^ It provides both local and global interpretability of prediction results. SHAP satisfies properties such as consistency and local accurac*y*, making it a robust and reproducible approach for assessing feature importance in complex models. In this study, we applied the SHAP package in Python to interpret the best‐performing classification model, obtaining SHAP values for each feature across all samples. Global feature importance was quantified by calculating the mean absolute SHAP value for each feature.

### 2.10 Transmission electron microscopy

Fresh tissues were fixed in 2.5% glutaraldehyde at 4°C for 12–24 h, rinsed with phosphate buffer (pH 7.35), and post‐fixed in 1% osmium tetroxide for 1–2 h. After washing, the samples were dehydrated through a graded ethanol and propylene oxide series, infiltrated with EMBed 812 resins, and polymerised at 60°C. Ultrathin sections (60–70 nm) were prepared using an ultramicrotome with a diamond knife, mounted on copper grids, and stained with uranyl acetate and lead citrate. The sections were then examined using a transmission electron microscope (HITACHI HT7800) at 80 kV.

### 2.11 Fluorescence in situ hybridisation

Tissues were fixed in in situ hybridisation fixative (Servicebio, G1113), routinely paraffin‐embedded, and sectioned at 4 µm. Sections were baked, deparaffinised, and rehydrated. Antigen retrieval was performed using a hot retrieval device at 90°C for 40 min, followed by digestion with proteinase K (Servicebio, G1234; 40°C, 5 min) and pre‐hybridisation. Sections were then hybridised with specific probes: Halomonas probe, 5′‐CCTGCTTTCTTCCTCAGAACGT‐3′, and Paenibacillus probe, 5′‐CCCAGGAGGGAAGATCGCGT‐3′, both at 1:100 concentration, using hybridisation buffer at 40°C. After hybridisation, sections were washed with graded SSC buffer (Servicebio, G3015), counterstained with DAPI (Servicebio, G1012), and mounted with antifade mounting medium (Servicebio, G1401). Fluorescence images were acquired using a Nikon ECLIPSE CI upright fluorescence microscope and Nikon DS‐U3 imaging system.

### 2.12 Cell culture

The human breast cancer cell lines MDA‐MB‐231 and MCF‐7 were purchased from the American Type Culture Collection (ATCC, USA). MDA‐MB‐231 cells were maintained in Dulbecco's Modified Eagle Medium (DMEM; Gibco, USA), and MCF‐7 cells were cultured in RPMI‐1640 medium (Gibco, USA). Both media were supplemented with 10% fetal bovine serum (FBS; Gibco, USA). Cells were incubated at 37°C in a humidified atmosphere containing 5% CO_2_.

### 2.13 Bacterial culture

*Paenibacillus pasadenensis* was purchased from the DSMZ collection, and *Halomonas hamiltonii* was kindly provided by Dr. Bing (Wuhan Institute of Virology, Chinese Academy of Sciences, China). *Paenibacillus pasadenensis* was revived from freeze‐dried powder in tryptic soy broth supplemented with 10 mg/L MnSO_4_·H_2_O and cultured at 30°C under aerobic conditions with shaking at 180–200 rpm. *Halomonas hamiltonii* was cultured in Luria–Bertani medium under the same conditions.

### 2.14 Bacterial infection

Activated *Paenibacillus pasadenensis* and *Halomonas hamiltonii* were suspended in antibiotic‐free DMEM supplemented with 10% FBS at a multiplicity of infection (MOI) of 50 and co‐cultured with a monolayer of breast cancer cells for 2–4 h.^22^, ^23^

### 2.15 Colony formation assay

Cells were seeded into six‐well plates and cultured in medium supplemented with 10% FBS for 10–14 days. Colonies were then fixed with formaldehyde solution and stained with crystal violet.

### 2.16 EdU proliferation assay

Cell proliferation was assessed using the BeyoClick™ EdU Cell Proliferation Kit (Beyotime, China). Briefly, 1 × 10^5^ cells were seeded into 24‐well plates and incubated according to the manufacturer's instructions. Proliferating cells were labelled with AF555, and nuclei were counterstained with Hoechst 33342. Images were captured under a fluorescence microscope, and the proliferation rate was calculated as the percentage of EdU‐positive cells relative to the total cell number.

### 2.17 Migration assay

For the migration assay, 8 × 10^4^ breast cancer cells were seeded into the upper chamber of a Transwell insert (Corning Incorporated, USA). The lower chamber was filled with DMEM or RPMI‐1640 medium supplemented with 20% FBS. After 48 h of incubation, cells were fixed with formaldehyde solution, stained with crystal violet, and examined under a light microscope.

### 2.18 Tools and databases

The following software tools were used in this study: QIIME2 (version 2020.6), FLASH (version 1.2.11), USEARCH (version 10.0), UCHIME (version 8.1), Trimmomatic (version 0.33), and RDP Classifier (version 2.2), GraphPad Prism (version 9.5.1). Analyses conducted in R software (version 4.3.0) utilised the following packages: ‘vegan’, ‘*ggClusterNet* package’. The following reference databases were employed: Silva rRNA gene database (http://www.arb‐silva.de); KEGG (https://www.kegg.jp/); and COG (https://www.ncbi.nlm.nih.gov/COG/).

## 3 RESULTS

### 3.1 Intratumoural microbiota differences between NNT and NDT group

The baseline characteristics of the NDT (*n* = 83) and NNT (*n* = 82) groups were comparable. The median age was 54 years in the NDT group and 55 years in the NNT group. In terms of pathological diagnosis, adenosis was the most common condition in both groups, followed by fibroadenoma, hyperplasia, and cysts (Supplementary Data 1a).

**FIGURE 2:**
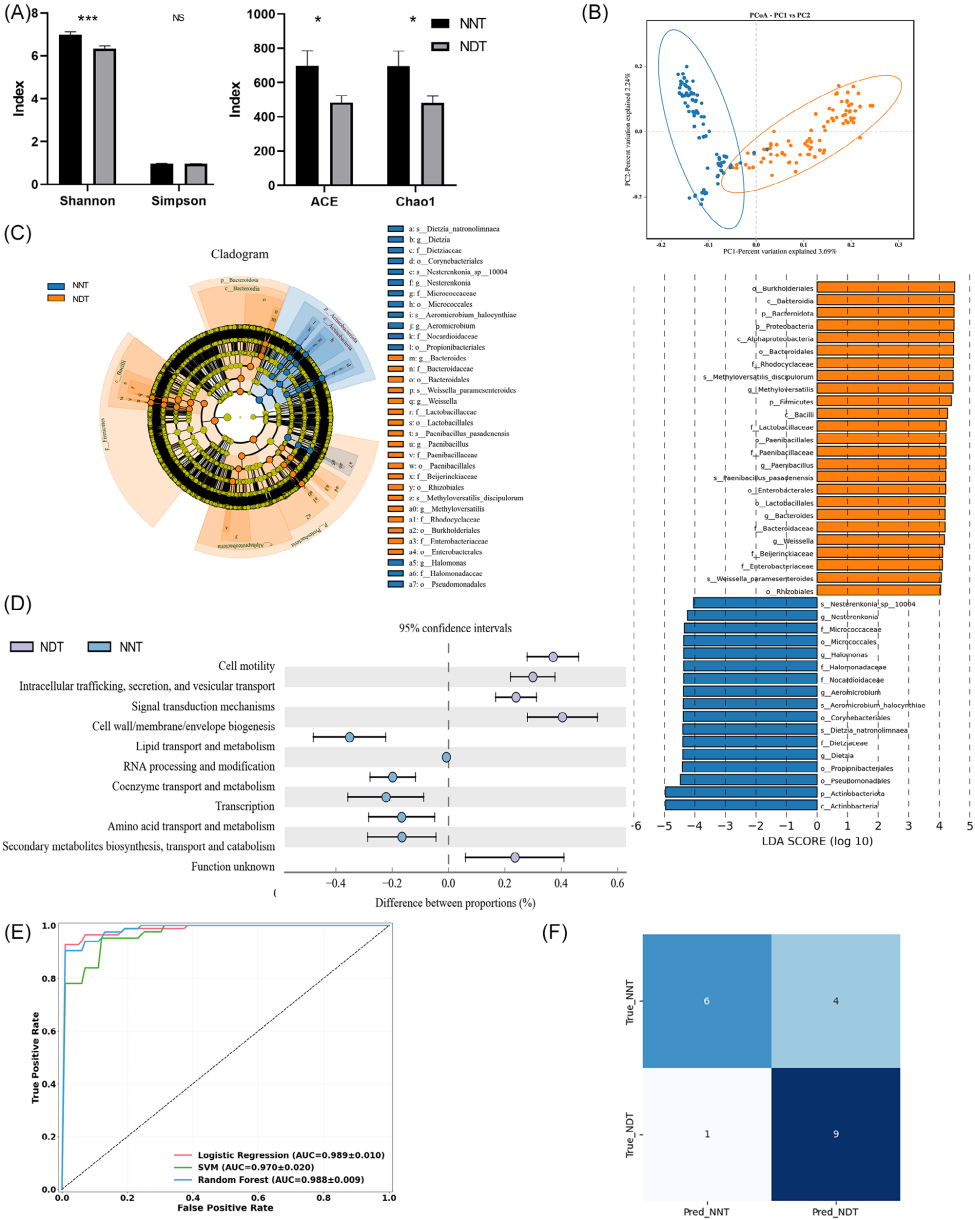
Multi‐dimensional comparison of tumoural microbiota between NNT and NDT groups. (A) α diversity indices between NNT and NDT groups. (B) β diversity between NNT and NDT groups. (C) Intratumoural microbial differences identified by LEfSe analysis between NNT and NDT groups(LDA > 4). (D) COG functional differences of intratumoural microbiota between NDT and NNT groups. (E) Performance of different machine learning models in predicting malignant transformation of breast nodules. (F) Confusion matrix of the external validation cohort. NDT, neoplastic development group; NNT, non‐neoplastic nodule group; RF, random forest; SVM, support vector machine; LR, logistic regression; AUC, area under the receiver operating characteristic curve. *p < .05; **p < .01, ***p < .001.

α diversity analysis revealed that the ACE index, Chao1 index, and Shannon index were all significantly lower in the NDT group compared with the NNT group (Figure 2A). β diversity analysis showed significant differences in microbial community composition between the two groups (*R* = .46, *p* = .001, Figure 2B), suggesting systematic divergence in the intratumoural microbiota. In the NNT group, the network was dense and stable (199 edges, 33 nodes, 5.5% negative edges). In the NDT group, the network became sparse (67 edges, 36 nodes) with more negative edges (22.4%), lower average degree (3.72), and clustering coefficient (.56), indicating reduced cooperation and increased competition before malignant transformation (Figure S1a and Supplementary Data 1b)

The dominant phyla in both groups were *Proteobacteria*, *Firmicutes*, *Bacteroidota*, and *Actinobacteriota*, accounting for approximately 80% and 90% of the total microbiota in the NDT and NNT groups, respectively (Figure S1b). Lefse analysis highlighted significant microbial differences between two groups, identifying biomarkers mainly from the *Bacteroidota*, *Firmicutes*, *Actinobacteriota*, and *Proteobacteria* phyla. Specifically, *Bacteroides*, *Weissella*, *Paenibacillus*, and *Methyloversatilis* were more abundant in the NDT group, whereas *Halomonas*, *Dietzia*, *Nesterenkonia*, and *Aeromicrobium* were enriched in the NNT group (LDA > 4) (Figure 2C). At the species level, taxa such as *Methyloversatilis discipulorum*, *Paenibacillus pasadenensis*, *Weissella cibaria*, and *Bosea vestrisii* were significantly enriched in the NDT group, whereas *Dietzia natronolimnaea*, *Aeromicrobium halocynthiae*, *Halomonas hamiltonii*, and *Nesterenkonia sp. 10004* were significantly decreased (all *p* < .05) (Figure S2, Supplementary Data 1d–h)

Functional pathway analysis showed significant differences between the groups in multiple KEGG and COG categories, particularly within ‘Metabolism’ pathways. The NNT group exhibited significantly higher enrichment scores for ‘amino acid metabolism’ and ‘lipid metabolism’ compared to the NDT group (both *p* < .05). In contrast, the NDT group showed relatively higher enrichment in ‘Glycan metabolism’ (*p* < .05) (Figures S1c and 2D, Supplementary Data 1i and j).

### 3.2 Dynamic changes of intratumoural microbiota during malignant transformation

**FIGURE 3:**
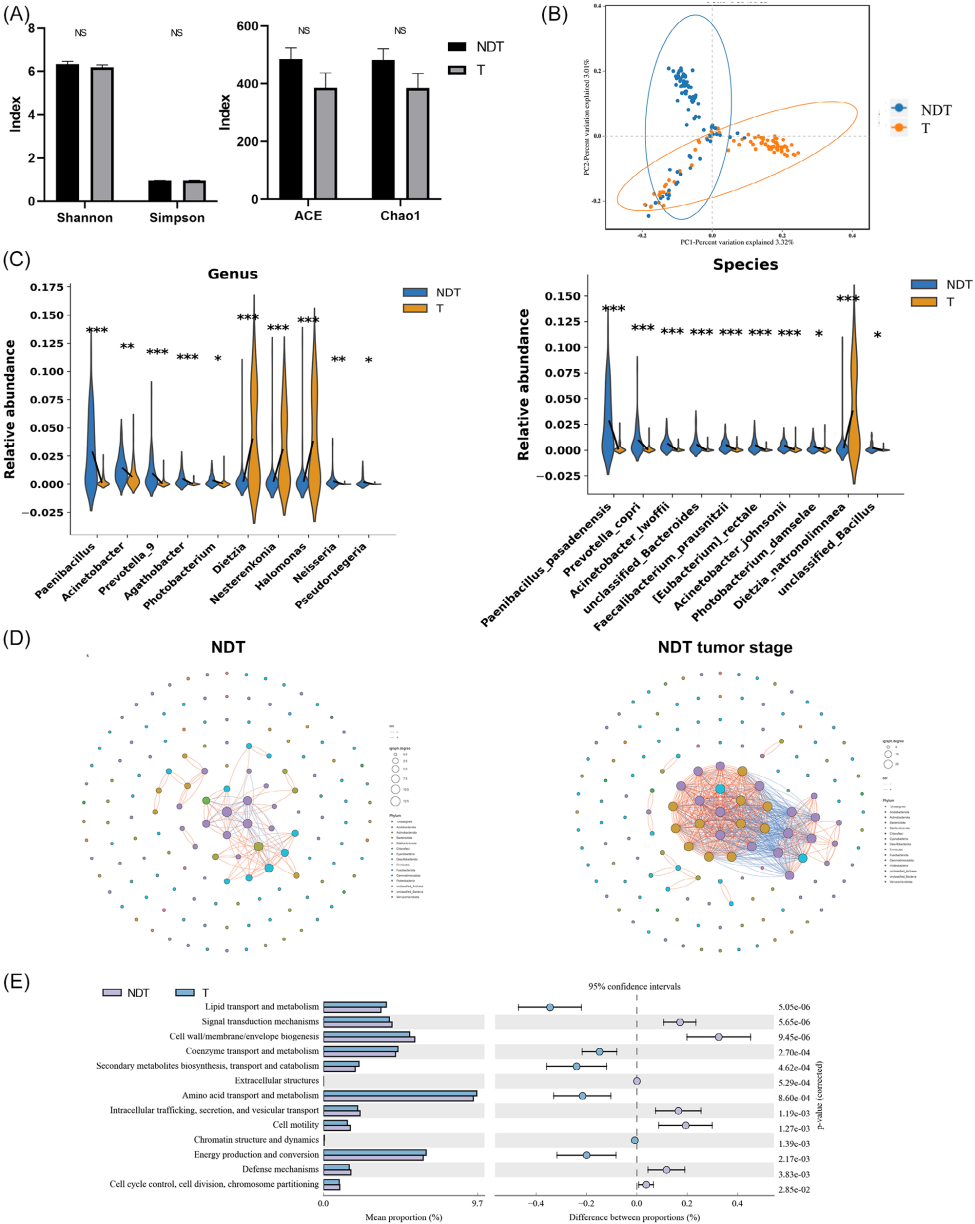
Dynamic changes of intratumoural microbiota during malignant transformation. (A) Changes in α diversity from benign (NDT) to malignant (Tumour) stage. (B) Changes in β diversity from benign (NDT) to malignant (Tumour) stage. (C) Intratumoural microbial dynamic changes from benign (NDT) to malignant (Tumour) stage. (D) Co‐occurrence network changes from benign (NDT) to malignant (Tumour) stage. (E) COG functional dynamic changes from benign (NDT) to malignant (Tumour) stage. NDT, neoplastic development group; T, tumour stage. *p < .05; **p < .01, ***p < .001.

To further elucidate the dynamic changes of intratumoural microbiota during the progression of benign breast nodules to malignant tumours, we performed 16S rRNA sequencing on paired tissue samples from the NDT group at both the benign nodule and subsequent malignant stages. No significant differences were observed in α diversity (Figure 3A). However, β diversity analysis revealed a significant difference in community structure between the two groups (*R* = .328, *p* = .001) (Figure 3B).

Building on these findings, we further analysed the taxonomic composition to identify specific microbial shifts associated with malignancy (Supplementary Data 2a–f). At the phylum level, the relative abundances of *Actinobacteriota* increased in tumour samples, whereas *Bacteroidota*, and *Firmicutes* markedly decreased. Further analysis at the family and order levels indicated significant enrichment of Gram‐positive Actinobacteria‐related taxa, such as *Dietziaceae*, *Nocardioidaceae*, and *Halomonadaceae*, in tumour tissues, whereas groups like *Bacteriovoracaceae* and *Paenibacillaceae* were reduced (all *p* < .05) (Figure S3a). At the genus and species levels, 33 genera and 49 species were identified as significantly different between benign and malignant stages. Genera including *Aeromicrobium*, *Dietzia*, *Halomonas*, and *Nesterenkonia* were significantly enriched in the tumour phase, while *Paenibacillus* and *Acinetobacter* were significantly decreased (all *p* < .05). Specifically, several species exhibited a marked upward trend during malignant transformation, including *Aeromicrobium halocynthiae*, *Delftia acidovorans*, *Dietzia natronolimnaea*, *Halomonas hamiltonii*, species of the *Nesterenkonia* genus, and *Nitriliruptor alkaliphilus*. In contrast, species such as *Acinetobacter lwoffii* and *Paenibacillus pasadenensis* were significantly decreased in malignant tissues (all *p* < .05) (Figures 3C and S3a).

Compared to nodules, the intratumoural microbial interaction network in tumours exhibits a marked increase in both nodes and edges, indicating a more complex ecological structure. (Figure 3D). Functional prediction analyses, based on both COG and KEGG annotations, consistently indicated that microbial communities in tumour tissues were significantly enriched in metabolic pathways such as ‘Amino acid metabolism’, accompanied by a relative reduction in ‘Glycan metabolism’ (*p* < .05) (Figures 3E and S3b, Supplementary Data 2g–h).

### 3.3 Intratumoural microbiota differences between BC and benign breast lesions

**FIGURE 4:**
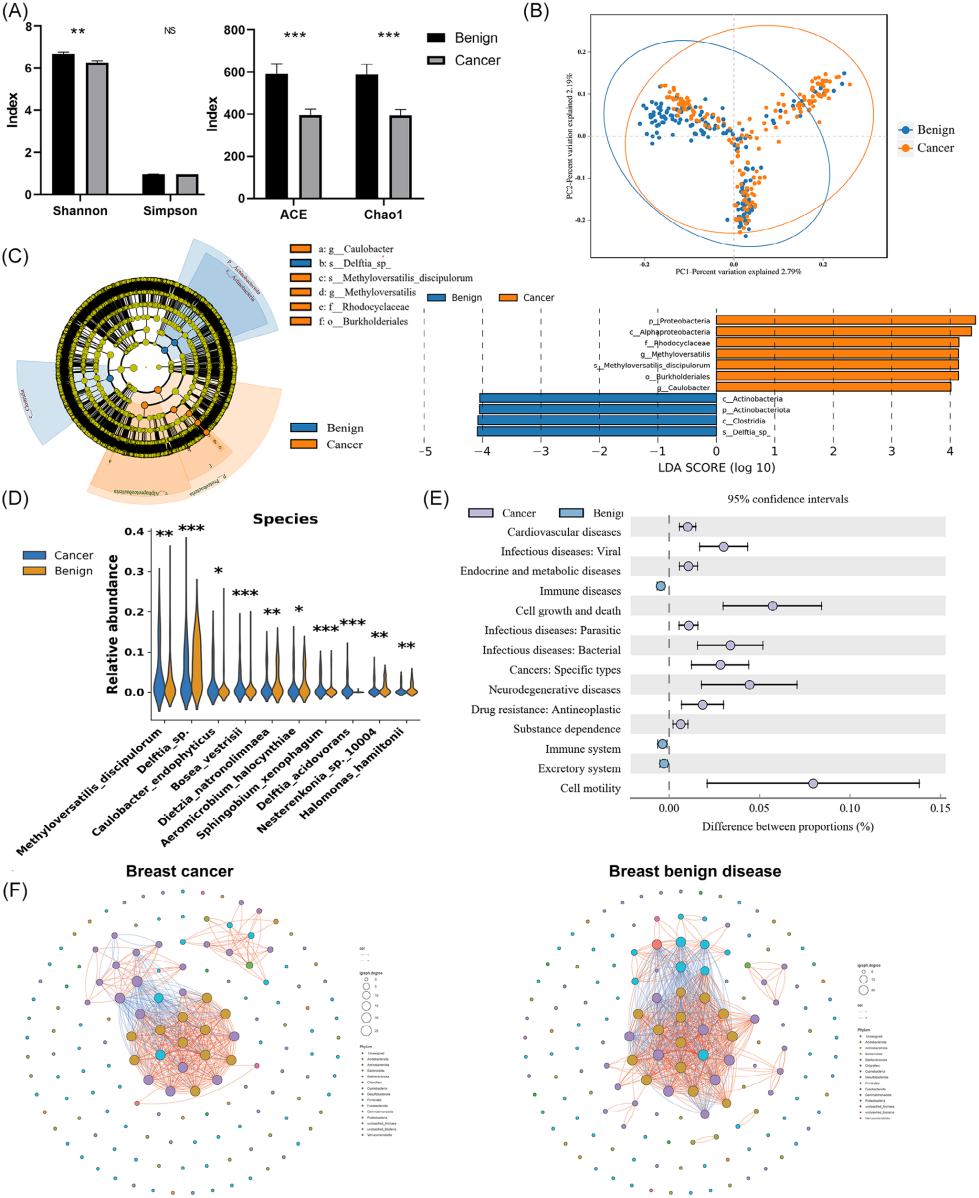
Multi‐dimensional comparison of tumoural microbiota between breast cancer and breast benign disease groups. (A) α diversity indices between cancer and benign groups. (B) β diversity between cancer and benign groups. (C) Intratumoural microbial differences identified by LEfSe analysis between cancer and benign groups(LDA > 4, *p* < .05). (D) Intratumoural microbial differences between cancer and benign groups at the species level. (E) KEGG functional differences of intratumoural microbiota between cancer and benign groups. (f) Co‐occurrence networks of cancer and benign groups. *p < .05; **p < .01, ***p < .001.

To systematically compare the differences in microbial communities between BC and benign breast lesions, we integrated data from 180 BC samples and 165 benign control samples for analysis. Results of α diversity analysis revealed that the BC group exhibited significantly lower Shannon (6.19 vs. 6.60), ACE (395.01 vs. 587.11), and Chao1 indices (393.14 vs. 584.80) compared to the benign group (all *p* < .01) (Figure 4A). β diversity analysis further demonstrated subtle differences in community structure between the two groups (Figure 4B).

Regarding microbial composition, BC tissues exhibited marked structural changes at multiple taxonomic levels (Figure 4C, Supplementary Data 3a–f). At the genus level, *Methyloversatilis*, *Weissella*, and *Bosea* were enriched in the tumour group, whereas *Delftia*, *Dietzia*, *Halomonas*, *Aeromicrobium*, and *Nesterenkonia* were significantly reduced (all *p* < .05). Species‐level analysis identified that *Methyloversatilis discipulorum*, *Bosea vestrisii*, and *Sphingobium xenophagum* were enriched in tumour tissues, while *Delftia sp*., *Dietzia natronolimnaea*, *Aeromicrobium halocynthiae*, and *Halomonas hamiltonii* were more abundant in benign lesions (all *p* < .05) (Figures 4D and S4).

In terms of occurrence network, the tumour group showed a smaller but more cooperative network (264 edges, 44 nodes, 76.9% positive), while the benign group had a larger, less coordinated network (363 edges, 51 nodes, 69.3% positive) (Figure 4F). Pathway enrichment analysis revealed that microbial communities in tumour tissues were significantly enriched in several functional pathways, including tumour‐related pathways, drug resistance to antineoplastic agents, cell motility, cell growth and death (Figure 4E, Supplementary Data 3g and h).

### 3.4 Correlations between intratumoural microbiota and clinical characteristics

After confirming the presence of microbial differences between BC and benign lesions, we further analysed microbiota characteristics across different clinical stages of BC (ductal carcinoma in situ [DCIS], stage I, stage II, and stage III) to explore the dynamic evolution of microbial communities during tumour progression. For the included 180 cases, 15 were diagnosed as DCIS, 34 were classified as Stage I, 58 as Stage II, and 73 as Stage III.

With tumour progression, the microbial network became simplified, with edges and nodes decreasing from 744/138 in DCIS to 135/58 in TNM‐III. The average degree declined (10.78 to 4.65), while the clustering coefficient slightly increased and edge density remained low. In TNM‐III, negative edges dropped to 6.7%, indicating weakened microbial competition in advanced tumours (Figure S5a, Supplementary Data 1b).

**FIGURE 5:**
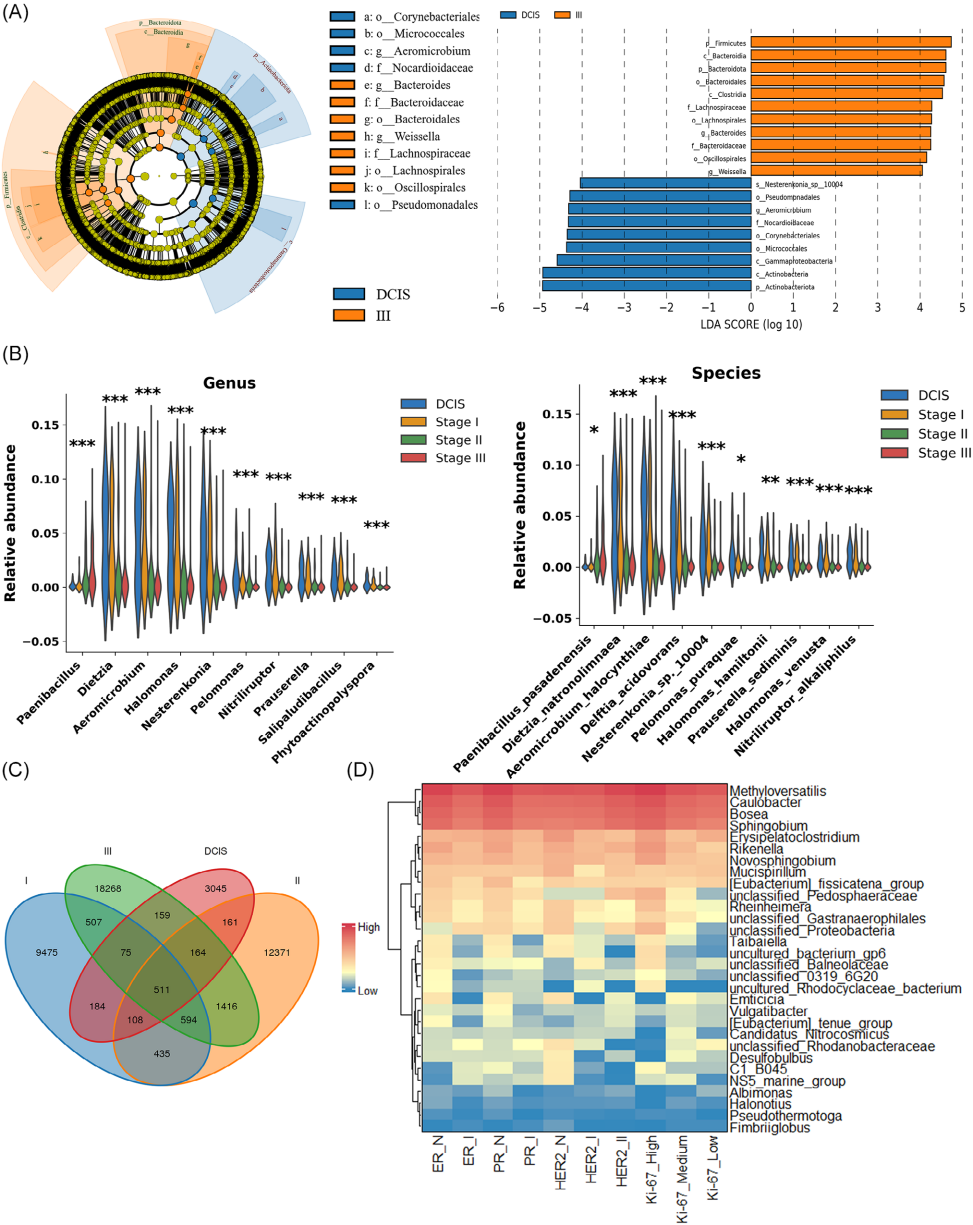
Correlations between Intratumoural microbiota and clinical characteristics. (A) Intratumoural microbial differences identified by LEfSe analysis among different stages (LDA > 4, *p* < .05). (B) Intratumoural microbial differences among different stages. (C) Shared and unique microbial features among breast cancer stages. (D) Heatmap of intratumoural microbiota across different clinical characteristics. DCIS, ductal carcinoma in situ; ER_N, ER‐negative; ER_I, ER‐positive; PR_N, PR‐negative; PR_I, PR‐positive; HER2‐N; HER2 (IHC 0); HER2‐I; HER2 (IHC +); HER2‐II; HER2 (IHC ++). *p < .05; **p < .01, ***p < .001.

Although overall diversity metrics did not exhibit significant changes, taxonomic composition analysis revealed stage‐associated trends. LEfSe analysis comparing microbial community differences across stages revealed significant discriminatory taxa between DCIS and stage III BC, indicating a ‘polarised’ shift in microbiota structure between early and advanced disease stages. Specifically, in stage III BC, the relative abundances of *Weissella* and *Bacteroides* were significantly increased compared to DCIS, whereas *Aeromicrobium* was notably enriched in DCIS samples (Figure 5A). Specifically, several taxa, including *Dietzia natronolimnaea*, *Aeromicrobium halocynthiae*, *Nesterenkonia sp. 10004*, *Halomonas hamiltonii*, and *Nitriliruptor alkaliphilus* (all *p* < .05), showed a gradual decline with advancing tumour stage, whereas *Paenibacillaceae pasadenensis* exhibited an increasing trend (Figures 5B and c and S5b, Supplementary Data 4a–f).

Building upon the analysis of stage‐associated microbiota changes, we further assessed correlations between microbial taxa and tumour molecular markers (Figure 5D). Fifteen cases diagnosed as DCIS and three cases with incomplete pathological information were excluded from the analysis. Overall, the microbial composition exhibited significant differences across various clinical subgroups. In the ER‐ and PR‐ groups, the relative abundances of *Methyloversatilis*, *Caulobacter*, *Sphingobium*, and *Rikenella* were markedly elevated, whereas these genera were less abundant in the ER+ and PR+ negative groups. In addition, in the HER2‐low expression, genera such as *Desulfobulbus*, *C1_B045*, *NS5_marine_group*, and *Albimonas* were significantly depleted compared to the HER2‐negative group. In the high‐proliferation Ki‐67 group, several genera showed significantly increased abundance, notably *Mucispirillum*, *Erysipelatoclostridium*, and *Taibaiell*. In contrast, the low‐proliferation group exhibited higher levels of *Desulfobulbus*, *Halonotius*, and *Pseudothermotoga*. In the high‐proliferation group, several genera, including *Mucispirillum*, *Erysipelatoclostridium*, and *Taibaiella*, exhibited significantly increased abundance. Conversely, the low‐proliferation group was characterised by higher levels of *Desulfobulbus*, *Halonotius*, and *Pseudothermotoga*.

### 3.5 Dynamic changes of the intratumoural microbiota during NAT

**FIGURE 6:**
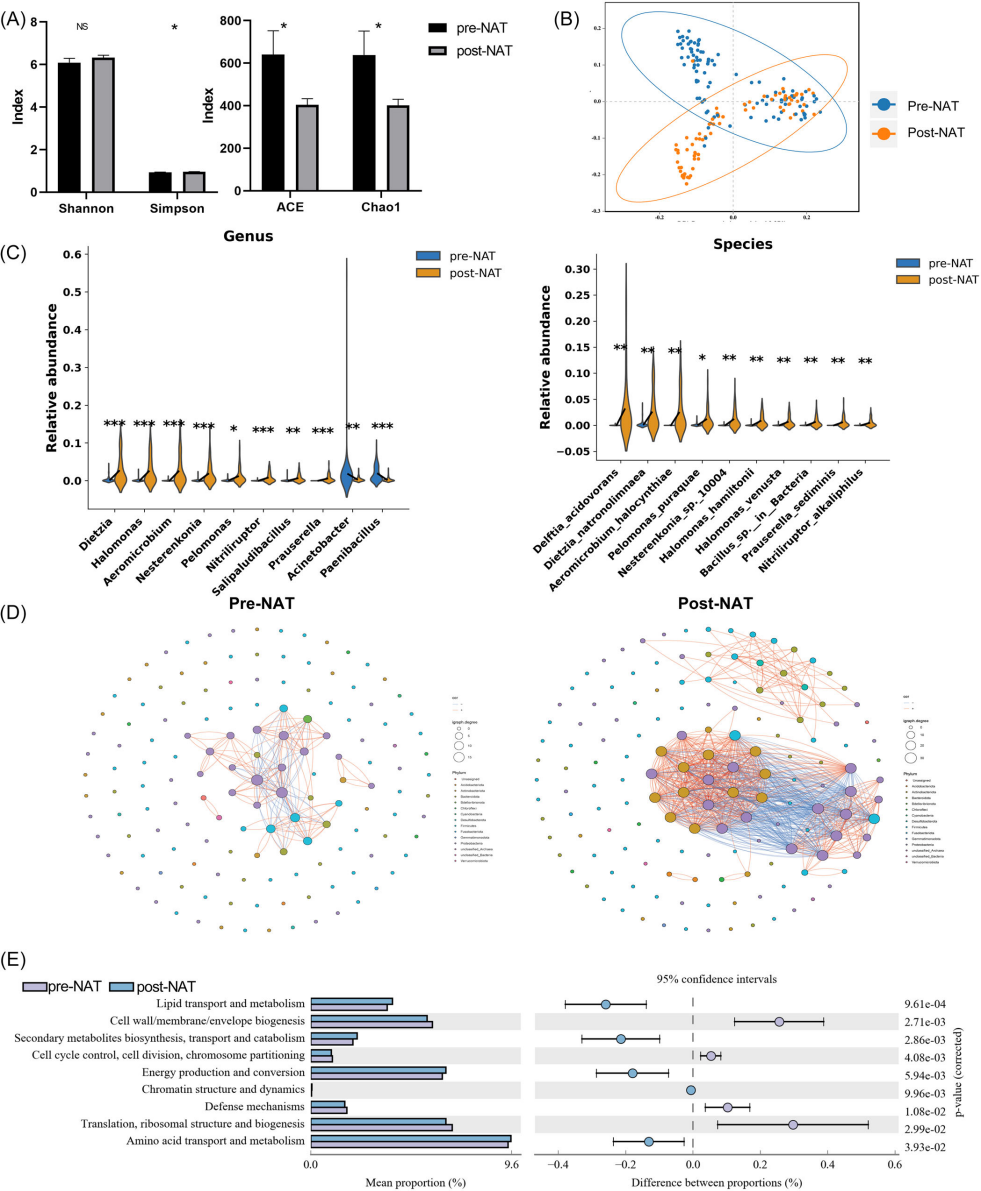
Dynamic changes of intratumoural microbiota during neoadjuvant therapy treatment. (A) Changes in α diversity during neoadjuvant therapy treatment. (B) Changes in β diversity during neoadjuvant therapy treatment. (C) Intratumoural microbial dynamic changes during neoadjuvant therapy treatment. (D) Co‐occurrence Network changes during neoadjuvant therapy treatment. (E) COG functional dynamic changes during neoadjuvant therapy treatment. pre‐NAT, preoperative neoadjuvant therapy; post‐NAT, postoperative neoadjuvant therapy. *p < .05; **p < .01, ***p < .001.

**FIGURE 7:**
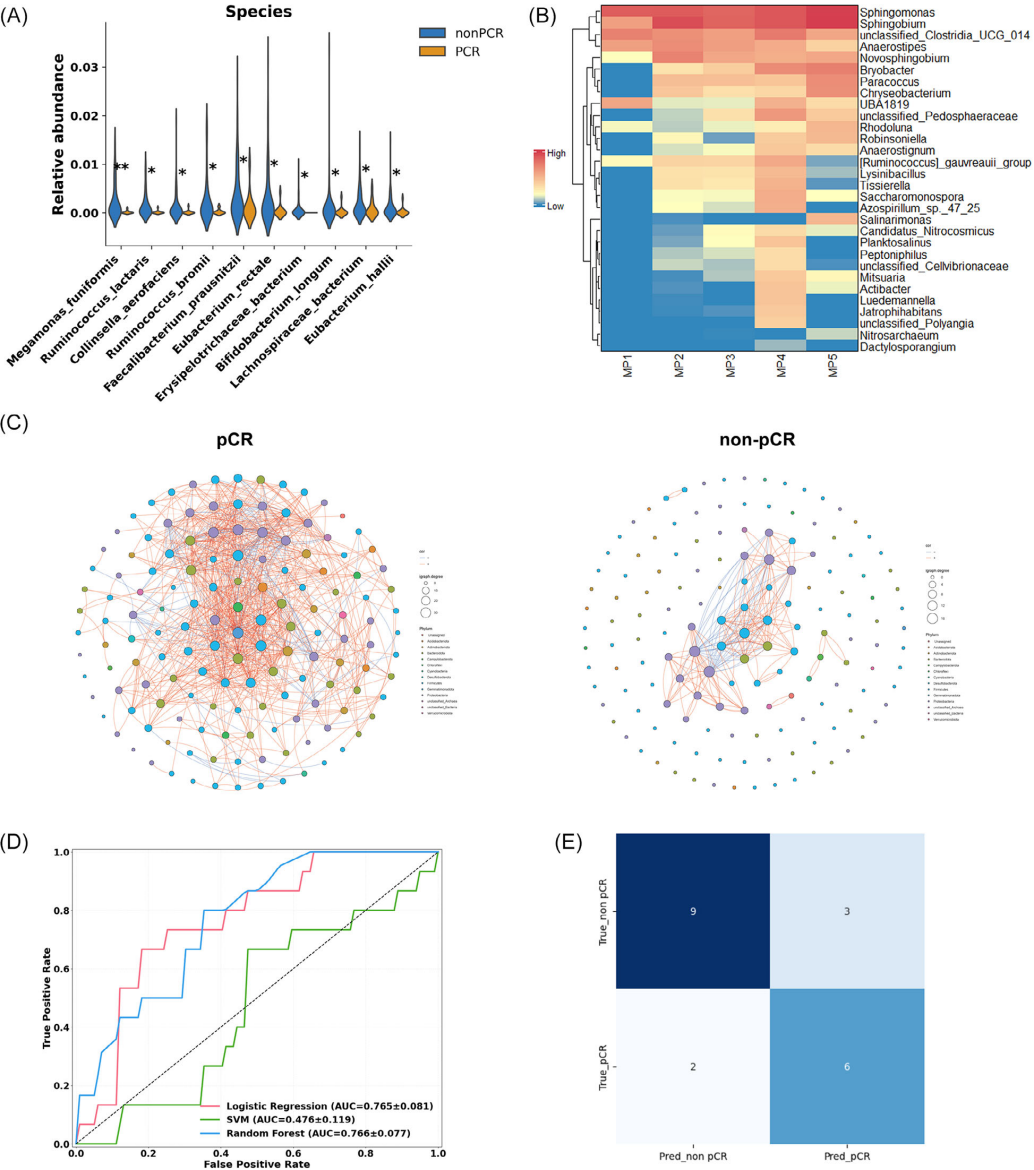
Multi‐dimensional comparison of tumoural microbiota between pCR and non‐pCR patients. (A) Intratumoural microbial differences between pCR and non‐pCR groups at the species level. (B) Heatmap of intratumoural microbiota across different Miller–Payne grades. (C) Co‐occurrence networks of pCR and non‐pCR groups. (D) Performance of different machine learning models in predicting neoadjuvant therapy response. (E) Confusion matrix of the external validation cohort. pCR, pathological complete response; non‐ pCR, non‐pathological complete response; MP, Miller–Payne; RF, random forest; SVM, support vector machine; LR, logistic regression; AUC, area under the receiver operating characteristic curve; SHAP, SHapley Additive exPlanations. *p < .05; **p < .01, ***p < .001.

Following NAT, α‐diversity indexes, including the Chao and ACE indices, significantly changed (Figure 6A). Additionally, β‐diversity analysis revealed a significant shift in microbial community composition (*R* = .21, *p* = .001) (Figure 6B) (Figure 7).

At the taxonomic level, notable changes were observed across multiple ranks (Supplementary Data 5a–f). At the phylum level, the relative abundances of *Actinobacteriota* increased post‐treatment, whereas *Firmicutes* decreased (all *p* < .05). At the genus and species levels, *Aeromicrobium*, *Halomonas*, *Dietzia*, *Nitriliruptor*, and *Nesterenkonia* genera, along with *Aeromicrobium halocynthiae*, *Halomonas hamiltonii*, *Dietzia natronolimnaea*, and *Nitriliruptor alkaliphilus* species, exhibited significant enrichment in the post‐NAT group. Conversely, the relative abundances of *Acinetobacter* and *Paenibacillus* were markedly reduced following treatment (all *p* < .05) (Figures 6C and S6a).

Following treatment, the number of network edges markedly increased (from 93 before NAT to 411 after), accompanied by an expansion of nodes to 62, while the average clustering coefficient remained stable, suggesting a re‐establishment of microbial ecological interactions (Figure 6D). Functional predictions based on KEGG and COG analyses demonstrated significant shifts in metabolic pathways after NAT (*p* < .05). Notably, pathways associated with amino acid metabolism, and lipid metabolism were significantly upregulated, whereas the functional abundance of glycan biosynthesis and metabolism pathways was substantially decreased (Figures 6E and S6b and Supplementary Data 5g and h).

### 3.6 Intratumoural microbiota differences between pCR and non‐pCR patients

To evaluate the potential association between intratumoural microbiota and therapeutic response, this study included 101 BC patients who underwent NAT. Patients were categorised into the pCR (*n* = 15) group and the non‐pCR (*n* = 86) group based on whether they achieved a pCR after surgery. There were no significant differences in age, Ki67, T stage, N stage, ER status, PR status, HER2 status, α diversity or β diversity between the two groups (*p* > .05) (Figure S7a and b, Supplementary Data 6a).

Microbial composition exhibited significant alterations across multiple taxonomic levels between the PCR and non‐PCR groups (Figure S7c and d, Supplementary Data 6b–g). LEfSe analysis revealed that Sphingobacteriales was significantly enriched in the PCR group, whereas Actinobacteriota showed higher abundance in the non‐PCR group. At the species level, *Ruminococcus lactaris*, *Megamonas funiformis*, *Faecalibacterium prausnitzii*, and *Eubacterium rectale* showed notable reductions in the PCR group compared to the non‐pCR group (all *p* < .05) (Figure 6A, Supplementary Data 6g). In addition, the pCR group exhibited a more complex network structure, with 640 edges and 139 nodes. Positive correlations accounted for 83.4% (534/640), higher than the non‐pCR group (81.4%, 83/102). Moreover, the pCR group showed higher average degree (9.2 vs. 5.23), clustering coefficient (.45 vs. .60), and relative modularity (.50 vs. .34; Figure 6C).

To explore the relationship between microbial composition and MP grade, we generated a genus‐level heatmap. The results revealed a clear gradient shift in microbial structure across MP grades. Genera such as *Sphingomonas*, *Sphingobiu*, and *Novosphingobium* were significantly enriched in the MP5 group. Notably, the intermediate MP3 and MP4 groups exhibited a transitional microbial profile. Genera such as *Ruminococcus_gauvreauii_group*, *Tissierella*, *Saccharomonospora*, and *Azospirillum_sp._47_25* showed increased abundance in these grades, indicating a possible shift in microbial community (Figure 6B).

### 3.7 Model for predicting nodule malignancy

To evaluate the potential of intratumoural microbiota and their metabolic pathways in predicting nodule malignant transformation, we constructed and compared three machine learning models. Among them, the random forest model demonstrated the best performance, achieving an AUC of .98 (Figure 2F). When applied to the external validation dataset of 20 patients, the model achieved an AUC of .84, indicating consistent predictive capability in an independent cohort.

To further elucidate the contribution of individual features to model predictions, we employed SHAP analysis. The results identified several key features influencing the model output, including the ‘Fatty acid degradation’, ‘Terpenoid backbone biosynthesis’, and ‘Valine, leucine and isoleucine degradation’ pathways, as well as specific microbial taxa such as *Prauserella, Salipaludibacillus, Nitriliruptor, Dietzia, Halomonas, Aeromicrobium* (Figure S1d)

### 3.8 Model for predicting neoadjuvant therapy response

Among the three evaluated models, RF demonstrated the highest performance, with an AUC of .77, surpassing that of LR model (AUC = .76) and the SVM model (AUC = .48) (Figure 6D). In the feature importance analysis of the predictive model for neoadjuvant therapy response, the top‐ranked variables included microbial metabolic pathways, bacterial taxa, and clinicopathological factors. Specifically, the most informative pathways were 2‐oxocarboxylic acid metabolism, phenylalanine/tyrosine/tryptophan biosynthesis, and arginine biosynthesis. At the taxonomic level, Subdoligranulum, Megamonas, and unclassified Clostridia UCG‐014 emerged as key microbial features. Among clinical parameters, Ki‐67 index, HER2 status, and age contributed substantially to the model (Figure S6e).

In the external validation cohort of 20 patients, the model achieved an AUC of .72. The confusion matrix showed 6 true positives, 3 false positives, 2 false negatives, and 9 true negatives, corresponding to an overall accuracy of 75% (Figure 6E).

### 3.9 Bacterial infection influences the proliferative and migratory capacities of breast cancer cell

**FIGURE 8:**
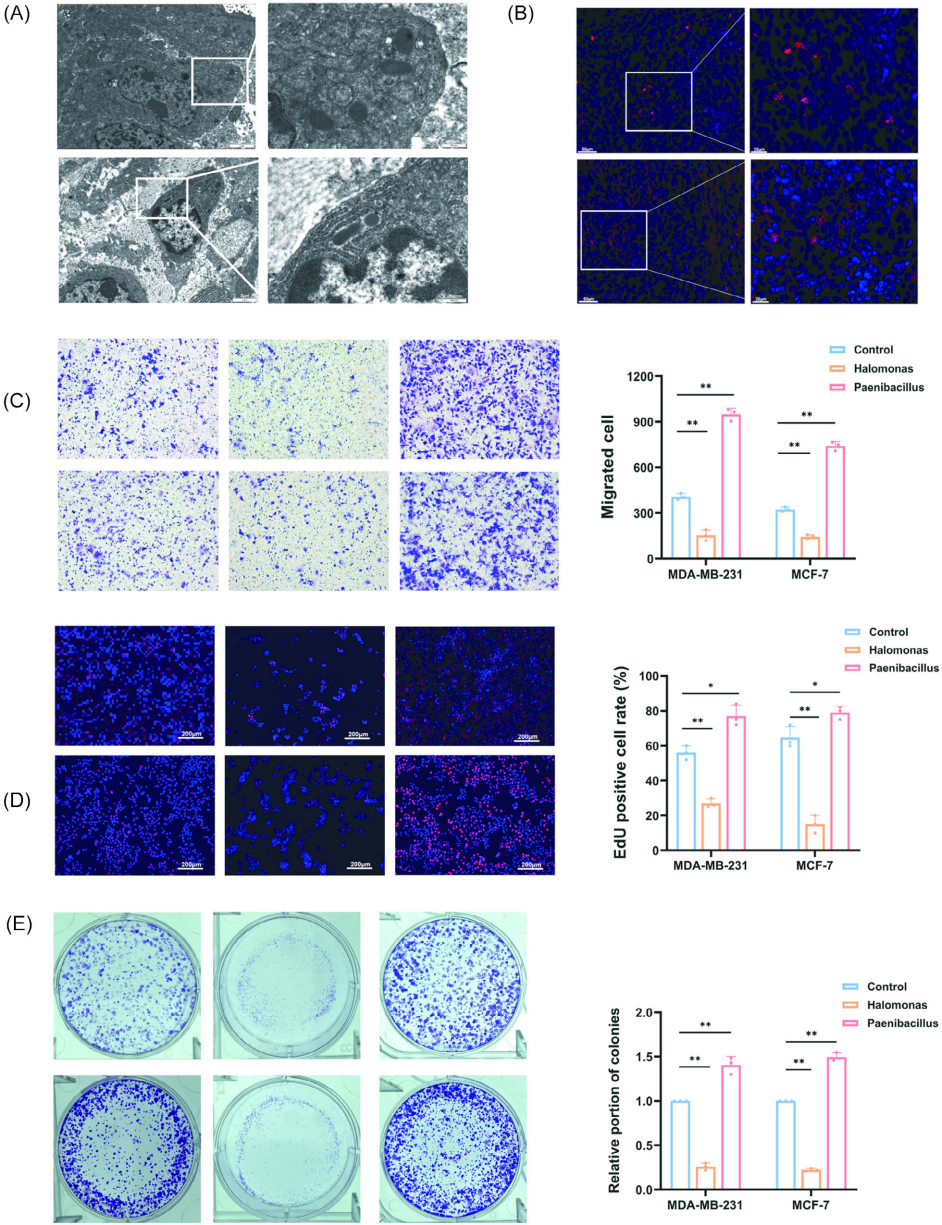
Bacteria influence the proliferative and migratory capacities of breast cancer cell lines. (A) Transmission electron microscopy (TEM) images showing bacteria in breast cancer tissues (scale bars: left, 2 µm; right, 500 nm). (B) Representative FISH staining images of bacteria in breast cancer tumours (scale bars: left, 50 µm; right, 20 µm). (C) Transwell migration assays and (D) EdU proliferation assays. (E) Colony formation assays performed in breast cancer cells infected with bacteria. *p < .05; **p < .01.

Transmission electron microscopy revealed the presence of *Paenibacillus pasadenensis* and *Halomonas hamiltonii* within breast cancer tissues, which was further confirmed by FISH analysis (Figure 8A and B). To explore the potential roles of these bacteria in breast cancer cells. MDA‐MB‐231 and MCF‐7 cell lines were infected with *Paenibacillus pasadenensis* or *Halomonas hamiltonii*. Transwell migration assays demonstrated that infection with *Halomonas hamiltonii* significantly reduced the migratory capacity of both breast cancer cell lines, whereas infection with *Paenibacillus pasadenensis* enhanced their migratory ability (Figure 8C). EdU proliferation assays further showed that *Halomonas hamiltonii* suppressed breast cancer cell proliferation, while *P. pasadenensis* promoted it (Figure 8D). Consistently, colony formation assays revealed a reduced clonogenic capacity in *Halomonas hamiltonii*‐infected cells, in contrast to an increased clonogenic potential following *Paenibacillus pasadenensis* infection (Figure 8E).

### 3.10 Microbial trajectories across tumour development and treatment

**FIGURE 9:**
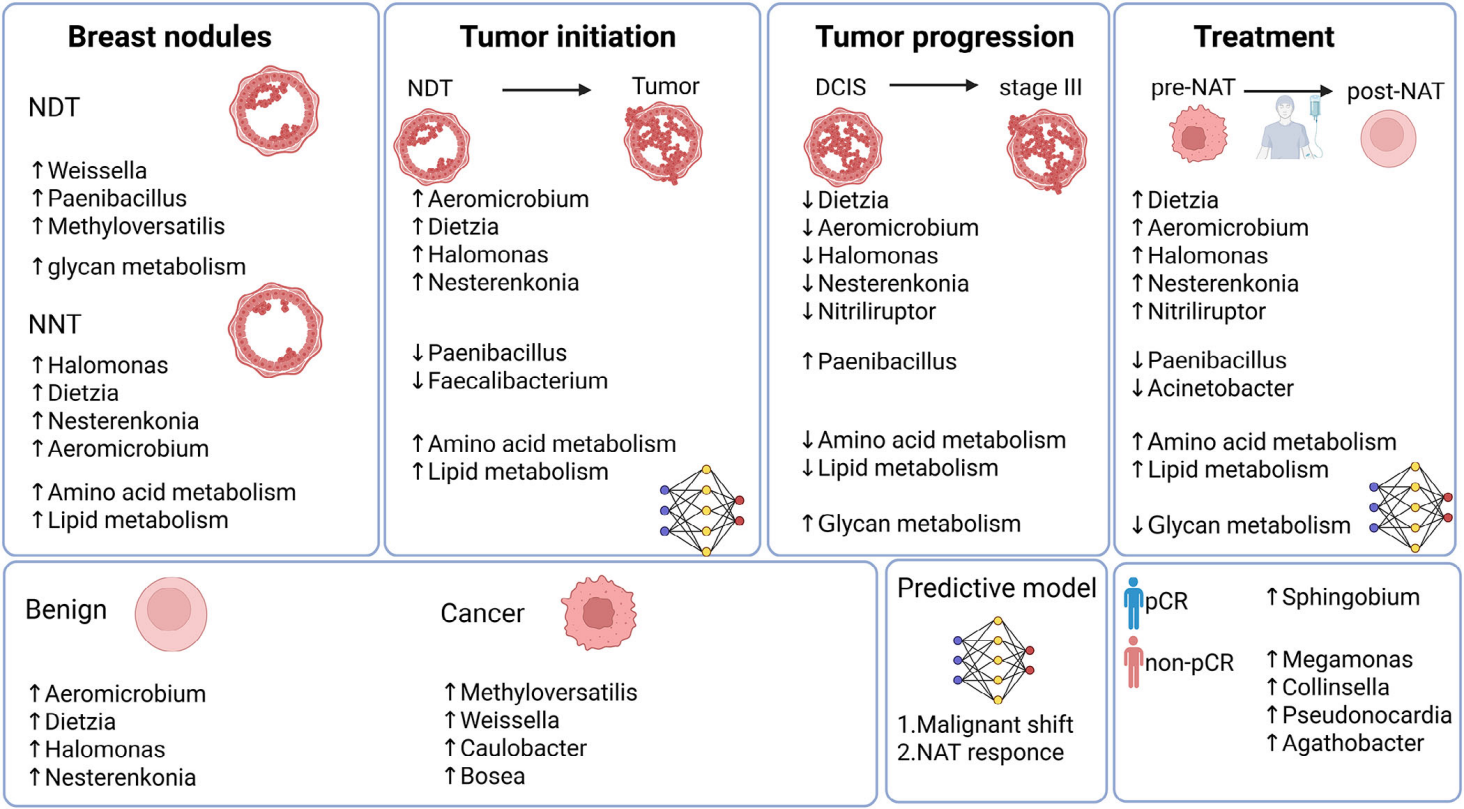
Graphical summary of the main findings and biological insights. NNT, non‐neoplastic nodule group; NDT, neoplastic development group; T, tumour stage; DCIS, ductal carcinoma in situ; pre‐NAT, preoperative neoadjuvant therapy; post‐NAT, postoperative neoadjuvant therapy; NAT, neoadjuvant therapy; pCR, pathological complete response; non‐ pCR, non‐pathological complete response.

Compared to NNT group, NDT group exhibited lower microbial diversity, with a relative enrichment of genera such as *Weissella*, *Paenibacillus*, and *Methyloversatilis*, whereas the NNT group showed higher abundances of *Halomonas*, *Dietzia*, *Nesterenkonia*, and *Aeromicrobium*. Functional analysis revealed that amino acid and lipid metabolism‐related pathways were significantly more active in the NNT group, while glycan metabolism pathways were enriched in the NDT group. During the malignant transformation of nodules, the abundances of *Aeromicrobium*, *Dietzia*, *Halomonas*, and *Nesterenkonia* markedly increased, whereas *Paenibacillus* significantly decreased. However, with advancing tumour stages, the relative abundances of *Dietzia*, *Aeromicrobium*, *Delftia*, *Nesterenkonia*, *Halomonas*, and *Nitriliruptor* gradually decline. Finally, following NAT, the microbial community composition underwent a notable reconstruction. After treatment, genera such as *Aeromicrobium*, *Halomonas*, *Dietzia*, *Nesterenkonia*, and *Nitriliruptor* showing significant increases in abundance, whereas *Paenibacillus* showed a marked decrease. Functional prediction analysis indicated enhanced activities in pathways related to amino acid metabolism and lipid metabolism, while pathways involved in glycan metabolism were diminished (Figure 9).

## 4 DISCUSSION

Our study firstly demonstrates that the malignant transformation of breast nodules and tumour progression are accompanied by significant changes in intratumoural microbial diversity and composition, with notable remodelling of the microbiota following neoadjuvant therapy. These key microbial taxa and their metabolic pathways may play important roles in breast cancer initiation, progression, and treatment response.

A class of candidate microbiota associated with reduced tumour progression was identified. Our experiments demonstrated, through functional assays, that *Halomonas hamiltonii* significantly inhibited tumour proliferation and activation. This observation aligns with previous studies indicating that *Halomonas* species possess antiproliferative properties and can suppress colony formation of MCF‐7 and DLD‐1 cancer cells through distinct mechanisms.^24^, ^25^ Specifically, sulphated polysaccharides derived from *Halomonas* have demonstrated significant antitumour effects by inhibiting cancer cell growth and metastasis, triggering apoptosis, and suppressing angiogenesis.^24^ Moreover, *Halomonas* species are known to produce pigments and exhibit strong antioxidant activity in murine macrophage cell lines (RAW264.7). *Halomonas* has also been shown to repair oxidative damage in hepatocellular carcinoma HepG2 cells induced by H_2_O_2_ exposure.^26^ Particularly, *Halomonas meridiana* has been found to promote apoptosis in multiple cancer cell lines, including HepG2, HeLa, LS 174T, and HCT 116, through the production of L‐glutaminase. L‐glutaminase facilitates the breakdown of L‐glutamine into L‐glutamate and ammonia, thereby depriving tumour cells of L‐glutamine, blocking de novo protein synthesis, and increasing oxidative stress, ultimately leading to selective inhibition of tumour growth.^27^ These findings lay the theoretical groundwork and suggest future research avenues for the application of *Halomonas* species in anticancer therapies.

The abundance of Delftia was notably decreased in tumour tissues compared to benign tissues, and further decreased with tumour progression. This finding is consistent with previous reports indicating that *Delftia* is markedly less abundant in breast tumour tissues relative to normal adjacent tissues.^28^ However, contrasting evidence has been observed in other malignancies; studies have reported a significant increase in *Delftia* abundance in pancreatic ductal adenocarcinoma and bladder cancer.^29^, ^30^ These divergent findings may reflect differences in tumour type and the associated microenvironment, suggesting that the involvement of *Delftia* in cancer development may be context‐dependent. In addition to well‐known bacterial genera, our study identified several less characterised taxa, including *Dietzia*, *Nesterenkonia*, and *Aeromicrobium*, which may play unique roles in BC. The specific pathogenic mechanisms underlying this association remain unclear and warrant further investigation.

In contrast to the tumour‐promoting microbial taxa discussed above, we also identified a significant role for *Paenibacillus* in BC initiation and progression, consistent with findings reported in other malignancies. This finding aligns with previous studies and further supports the potential role of *Paenibacillus* in the breast tumour microenvironment. Prior studies have shown that increased abundance of *Paenibacillus* in BC patients is associated with poor survival, with patient survival probability dropping to approximately 30%.^31^ These observations suggest that *Paenibacillus* may promote tumour development or progression by altering the tumour microenvironment, enhancing inflammatory responses, or facilitating metabolic pathways that create favourable conditions for tumour cell growth.

Our study also observed that *Methyloversatilis discipulorum* was more prevalent in the NDT group than in the NNT group, and significantly decreased after neoadjuvant therapy. Compared with normal tissues, *Methyloversatilis discipulorum* has been shown to be significantly upregulated in lung adenocarcinoma and head and neck cancer, with its abundance negatively correlated with OS and DFS.^32^, ^33^ Further analyses revealed a positive correlation between *Methyloversatilis discipulorum* abundance and the expression of Golgi membrane protein 1 (GOLM1).^33^ GOLM1 is involved in promoting tumour immune evasion and metastasis by attracting myeloid‐derived suppressor cells. Additionally, GOLM1 enhances the stability of PD‐L1 and facilitates its transport to tumour‐associated macrophage‐derived exosomes, thereby suppressing CD8+ T cell function and contributing to an immunosuppressive tumour microenvironment.^32^ Interestingly, *Methyloversatilis discipulorum* may also be involved in modulating epigenetic regulation via participation in SETD8‐mediated methylation of histone H4.^33^

Beyond their involvement in immune regulation and signalling pathways, intratumoural microbiota may also influence tumour progression and therapeutic responses by reshaping the metabolic landscape of the TME.^34^, ^35^ Our functional pathway analyses indicated that glycolysis was significantly enhanced in BC tissues, whereas amino acid and lipid metabolism pathways were relatively suppressed. Following therapy, glycolytic activity decreased, while amino acid and lipid metabolism were upregulated. This dynamic shift is consistent with patterns observed in other tumour types, suggesting a close relationship between metabolic status, tumour biological behaviour, and therapeutic responses. Amino acids not only serve as building blocks for protein synthesis but also participate in signal transduction and immune regulation. Recent findings have demonstrated that microbiota‐mediated modulation of amino acid metabolism – such as glutamine and arginine metabolism – can significantly impact tumour metabolism and immune cell function, ultimately shaping the immune microenvironment.^36^, ^37^ Glycolysis, as a classic form of metabolic reprogramming, plays a prominent role in tumour–microbiota interactions. Certain intratumoural bacteria can enhance glycolytic flux in cancer cells, exacerbating the Warburg effect and promoting an acidic TME that fosters immune suppression.^38^, ^39^, ^40^ Notably, *Fusobacterium nucleatum* has been shown to strengthen glucose metabolism by regulating key metabolic factors such as GLUT1 and ENO1, even influencing radiotherapy sensitivity.^41^, ^42^ Regarding lipid metabolism, microbiota may modulate the expression of key enzymes such as fatty acid synthase (FASN), thereby promoting de novo lipid synthesis to enhance membrane construction and signalling capabilities in cancer cells. For instance, nuclear *Fusarium* species have been demonstrated to induce FASN expression, a critical enzyme in colorectal cancer cell lipid biosynthesis.^43^ This metabolic aberration not only supports tumour cell proliferation but is also linked to chemotherapy resistance.

Microorganisms exert critical immunomodulatory effects within TME through their secreted metabolites. For example, short‐chain fatty acids such as butyrate can induce the differentiation of Foxp3⁺ regulatory T cells while inhibiting dendritic cell maturation, thereby establishing an immunosuppressive microenvironment.^44^ These microbial metabolites can also promote tumour cell autophagy and drive the polarisation of M2 macrophages within the TME, further reinforcing its suppressive characteristic.^44^, ^45^ Key bacterial taxa identified in our study, including *Methyloversatilis discipulorum*, may contribute to immune evasion and tumour progression by upregulating GOLM1, recruiting MDSCs, and enhancing PD‐L1 stability and transport, ultimately inhibiting CD8⁺ T cell function.^33^ *Paenibacillus* may facilitate tumour growth by modulating inflammatory responses and metabolic pathways. In contrast, microbes such as *Halomonas* and *Aeromicrobium* appear to suppress tumour development through antioxidant production, induction of tumour cell apoptosis, and remodelling of the metabolic environment.^27^ Furthermore, microbes may influence tumour immunity via additional mechanisms, including complement system regulation and cytokine secretion. Collectively, these findings indicate that microorganisms and their secreted metabolites play a central role in shaping the TME, regulating immune responses, and influencing tumour initiation and progression, providing a theoretical basis for future microbiome‐targeted cancer interventions.

Our findings reveal that during the malignant transformation and subsequent tumour progression, the intratumoural bacterial communities undergo dynamic and fluctuating changes. Compared with NNT, NDT exhibited significantly lower abundances of genera such as *Halomonas*, *Dietzia*, *Nesterenkonia*, and *Aeromicrobium*. However, during the nodule‐to‐tumour transition, the abundances of these genera increased markedly. Integrative analysis further indicated that overall, these bacterial taxa were less abundant in malignant breast tissues compared to benign tissues and continued to decline with advancing tumour stage. This dynamic pattern, characterised by an initial increase followed by a progressive decrease, appears paradoxical. We propose several possible explanations for this phenomenon. First, the malignant transformation samples in this study predominantly represented carcinoma in situ or early‐stage BC. Thus, the observed transient increase in bacterial abundance may reflect early microecological remodelling associated with tumour initiation. These bacteria might temporarily expand during the early phases of tumour development, potentially participating in local microenvironment regulation by mechanisms such as immune activation or metabolic competition, and possibly even attempting to counteract tumourigenesis.^46^ Second, as the tumour progresses to more advanced stages, profound alterations in the tumour microenvironment – including hypoxia, immune suppression, and metabolic dysregulation – may create conditions that are no longer favourable for the survival and proliferation of these potentially beneficial bacteria, leading to their gradual decline.^47^ Simultaneously, such changes could facilitate the expansion of pathogenic or tumour‐promoting bacteria, thereby further driving tumour progression. We therefore propose that this ‘rise‐then‐fall’ dynamic may reflect a biphasic role of the intratumoural microbiota during tumour development: certain bacterial taxa may initially increase to support local tissue homeostasis during malignant transformation but are subsequently suppressed as the tumour microenvironment deteriorates. Further studies are needed to elucidate the specific functions of these bacterial taxa and their interactions with the host immune system.^48^

Importantly, this study also explores potential clinical value of intratumoural microbiota in risk prediction and therapeutic decision‐making. In the predictive model for neoadjuvant therapy response, the absolute SHAP values of individual features were all below .2 – a phenomenon commonly observed in high‐dimensional and sparse microbiome datasets. Although the contribution of any single taxon to the model outcome may be modest, their collective and synergistic effects can meaningfully influence the model's decision‐making process. Moreover, SHAP values reflect the relative importance of features within the model rather than their absolute effect size. Although traditional imaging modalities and histopathological evaluation can detect existing lesions, they lack prospective predictive power regarding future malignant transformation. In contrast, our microbiota‐based model achieved high predictive performance in the external validation cohort (AUC = .84), suggesting that microbiota structural alterations may precede morphological changes and thus have potential as early biomarkers for risk identification. Second, we established a model to predict response to NAT, further investigating the potential utility of microbiota signatures in guiding treatment strategies. This model also demonstrated excellent performance in distinguishing responders from non‐responders (AUC = .72), indicating that intratumoural microbiota may be closely associated with treatment sensitivity. Recent studies have revealed that the tumour‐associated microbiome can modulate therapeutic response by influencing the immune microenvironment, drug metabolism, and inflammatory signalling pathways.^48^ The microbiota signatures we identified may serve as future predictive markers or therapeutic modulators, offering new perspectives for personalising NAT regimens.

Despite providing valuable insights into the dynamic changes of the intratumoural microbiota during BC initiation, development, and treatment, and establishing predictive models with potential clinical utility, there are several limitations in this study. First, while we characterised shifts in the microbial community structure, further studies are needed to clarify the functional contributions of specific bacterial taxa to tumour progression. Second, multi‐omics integration was not feasible in this study due to the lack of matched transcriptomic and metabolomic data. This limitation hinders a deeper mechanistic understanding of the host–microbiome interactions and the functional consequences of microbial alterations. Future studies incorporating paired host and microbial omics data will be valuable for elucidating causal pathways. Finally, although our predictive models demonstrated promising clinical application potential, they were developed and validated using retrospective data. Prospective, large‐scale, multi‐centre cohort studies are necessary to further evaluate the reliability, generalisability, and clinical translational value of these microbiota‐based predictive tools.

## 5 CONCLUSION

This study revealed significant dynamic changes in the intratumoural microbiota during BC initiation, progression, and treatment. We identified Paenibacillus as potentially tumour‐promoting bacteria, while Halomonas, Delftia, Dietzia, Nesterenkonia, and Aeromicrobium were observed to be linked with contrasting associations. These findings highlight the close relationship between microbiota alterations and BC clinical stages, suggesting that specific bacterial shifts may influence tumour development through modulation of the tumour microenvironment, offering novel insights for precision oncology and microbiome‐based interventions in breast cancer.

## AUTHOR CONTRIBUTIONS

L.Q. and Y.P. conceived and designed the study, performed data analysis, and drafted the manuscript. L.Q. and H.R. contributed to microbiome data interpretation and manuscript revision. Z.Y, J.Y, and D.S. participated in data collection. L.Q, J.Z., and J.X. were responsible for clinical data acquisition and sample handling. S.C. assisted in literature review and figure preparation. M.S. performed the experiments. P.Y. supervised the overall project, critically revised the manuscript, and approved the final version for submission.

## CONFLICT OF INTEREST STATEMENT

The authors declare no conflicts of interest related to this study.

## ETHICS STATEMENT

This research was conducted in line with ethical standards and received approval from the Ethics Committee of the Cancer Hospital, Chinese Academy of Medical Sciences (Approval No. NCC2025C‐626). Written informed consent was obtained from all patients prior to sample collection.

## Supporting information



Supporting Information

Supporting Information

Supporting Information

Supporting Information

Supporting Information

Supporting Information

Supporting Information

Supporting Information

Supporting Information

## Data Availability

The datasets supporting the findings of this study are available from the corresponding author upon reasonable request.
