# Supplementary figures and images for: Dynamics of the intratumoural microbiome across malignant transformation and treatment in breast cancer
Source: Clin Transl Med. 2025 Oct 1;15(10):e70492. doi: 10.1002/ctm2.70492 (PMC12485822; doi:10.1002/ctm2.70492)

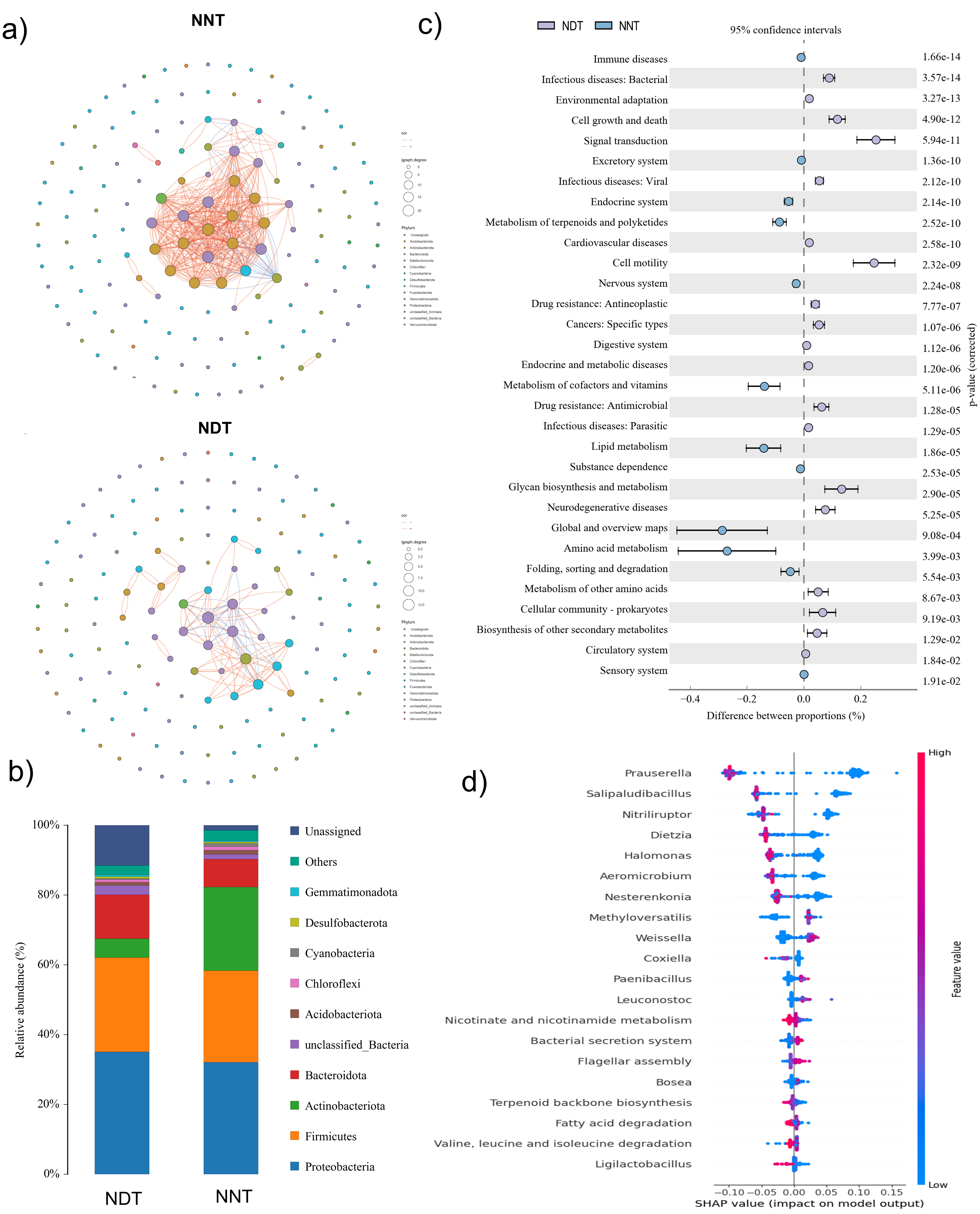

Supplement: Supplementary file 1 — Supporting Information [file CTM2-15-e70492-s008.TIF]

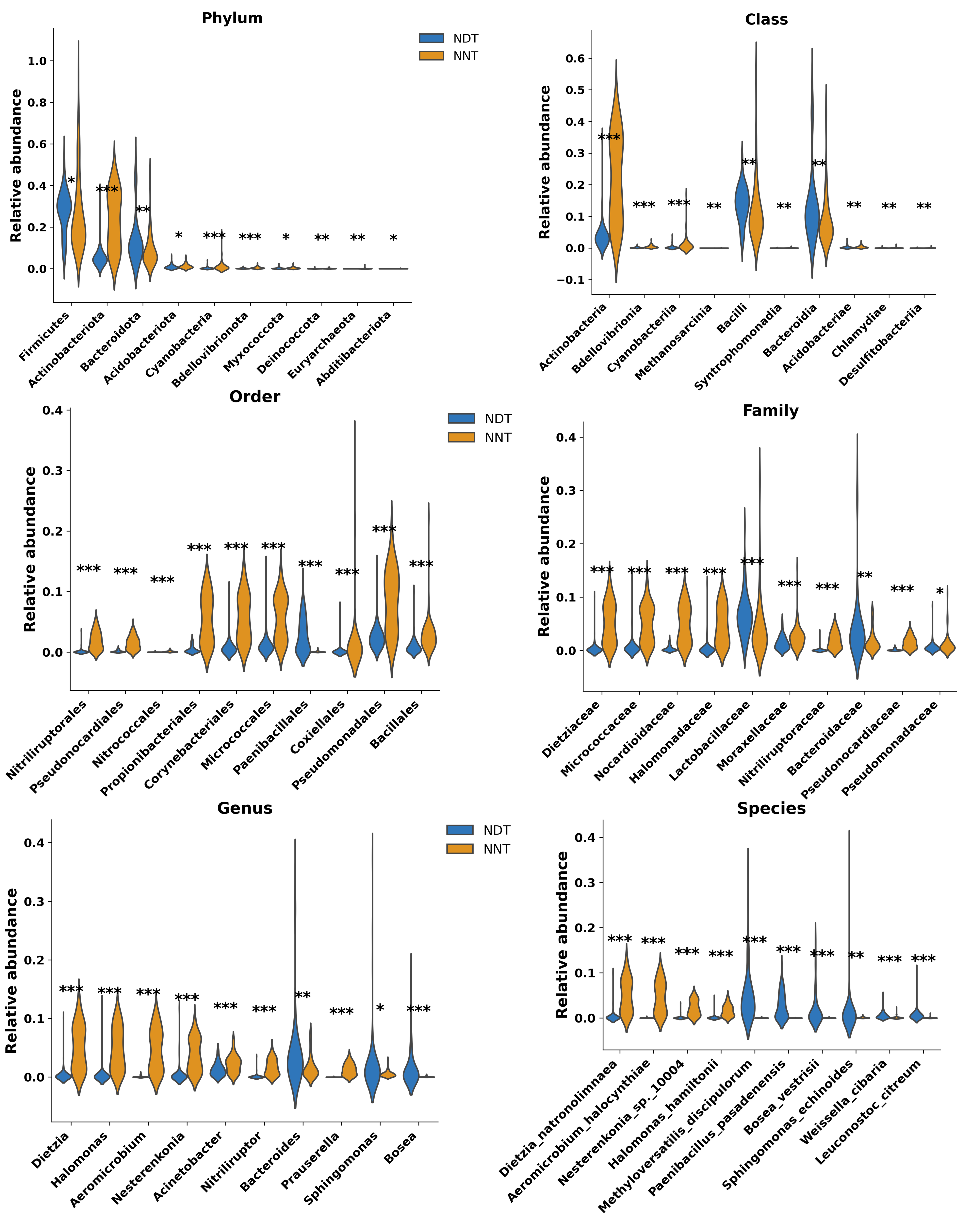

Supplement: Supplementary file 2 — Supporting Information [file CTM2-15-e70492-s002.tif]

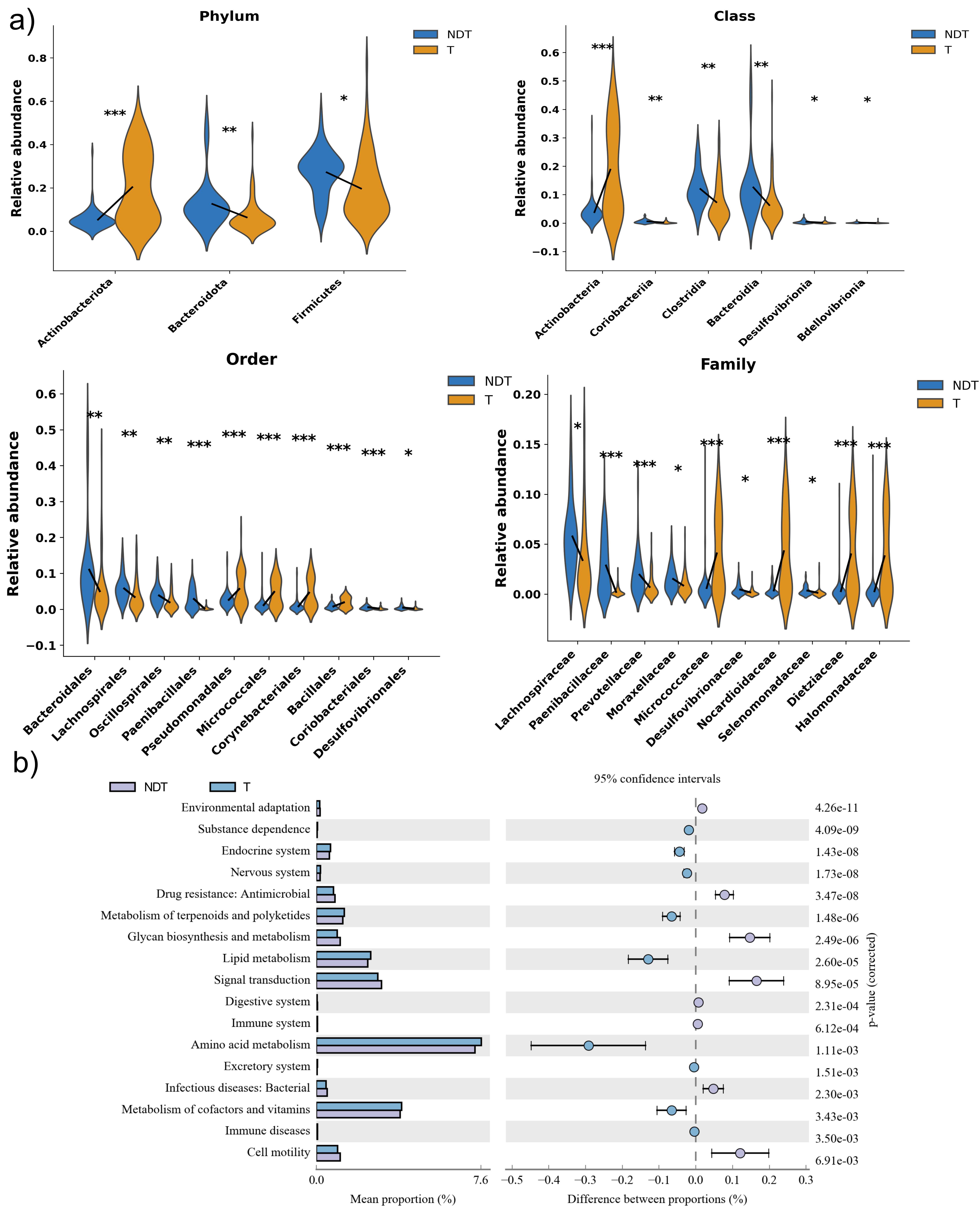

Supplement: Supplementary file 3 — Supporting Information [file CTM2-15-e70492-s001.TIF]

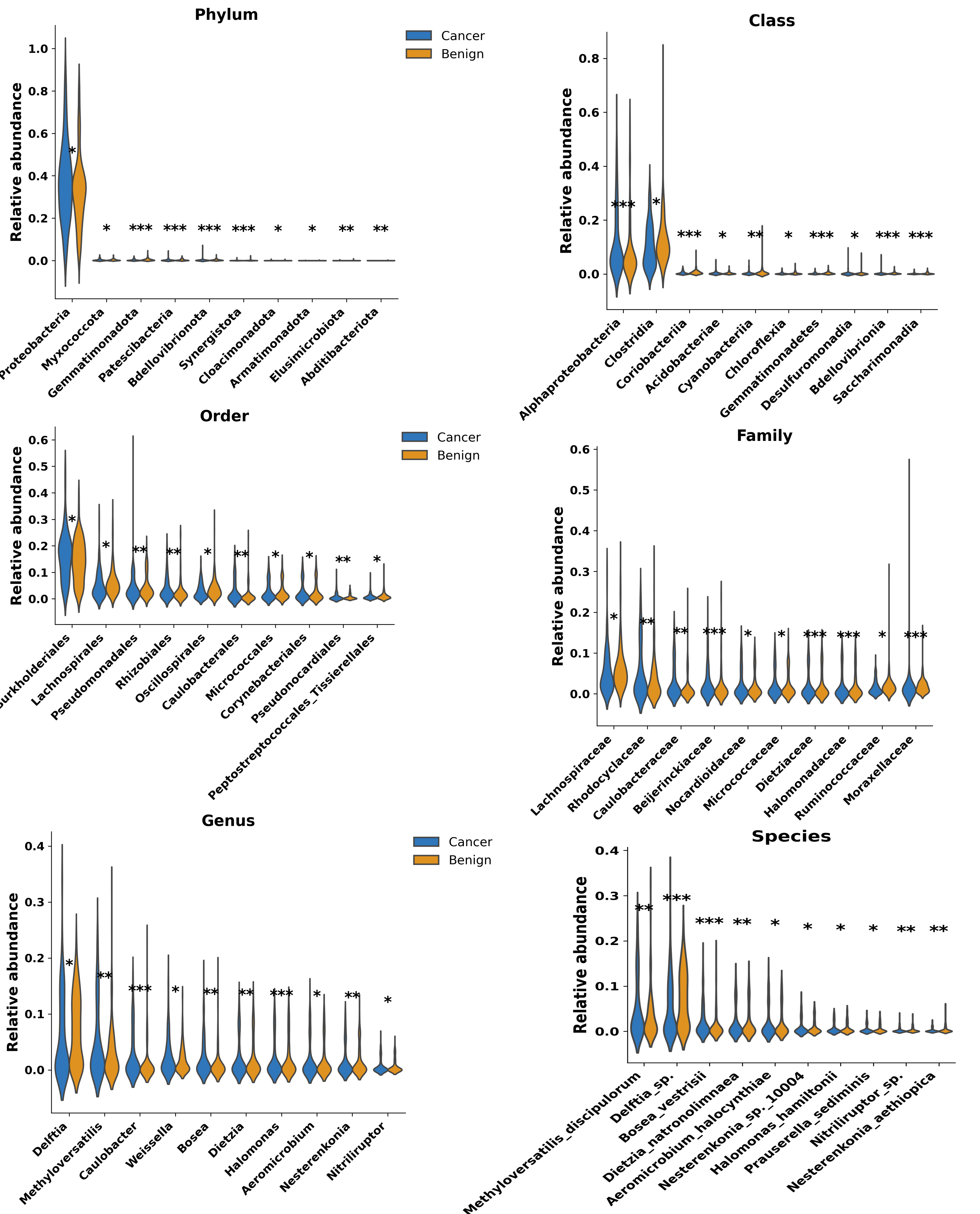

Supplement: Supplementary file 4 — Supporting Information [file CTM2-15-e70492-s003.TIF]

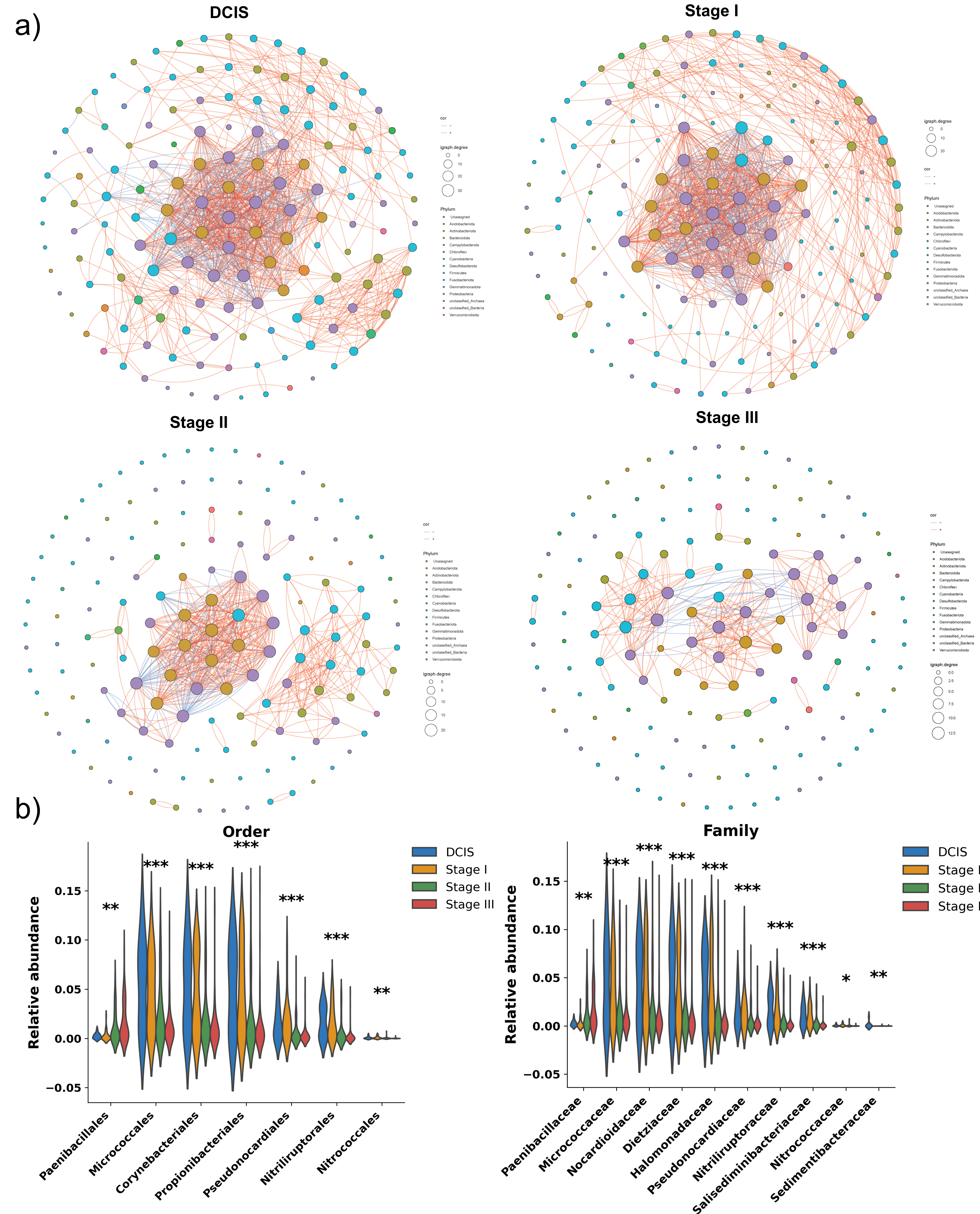

Supplement: Supplementary file 5 — Supporting Information [file CTM2-15-e70492-s004.tif]

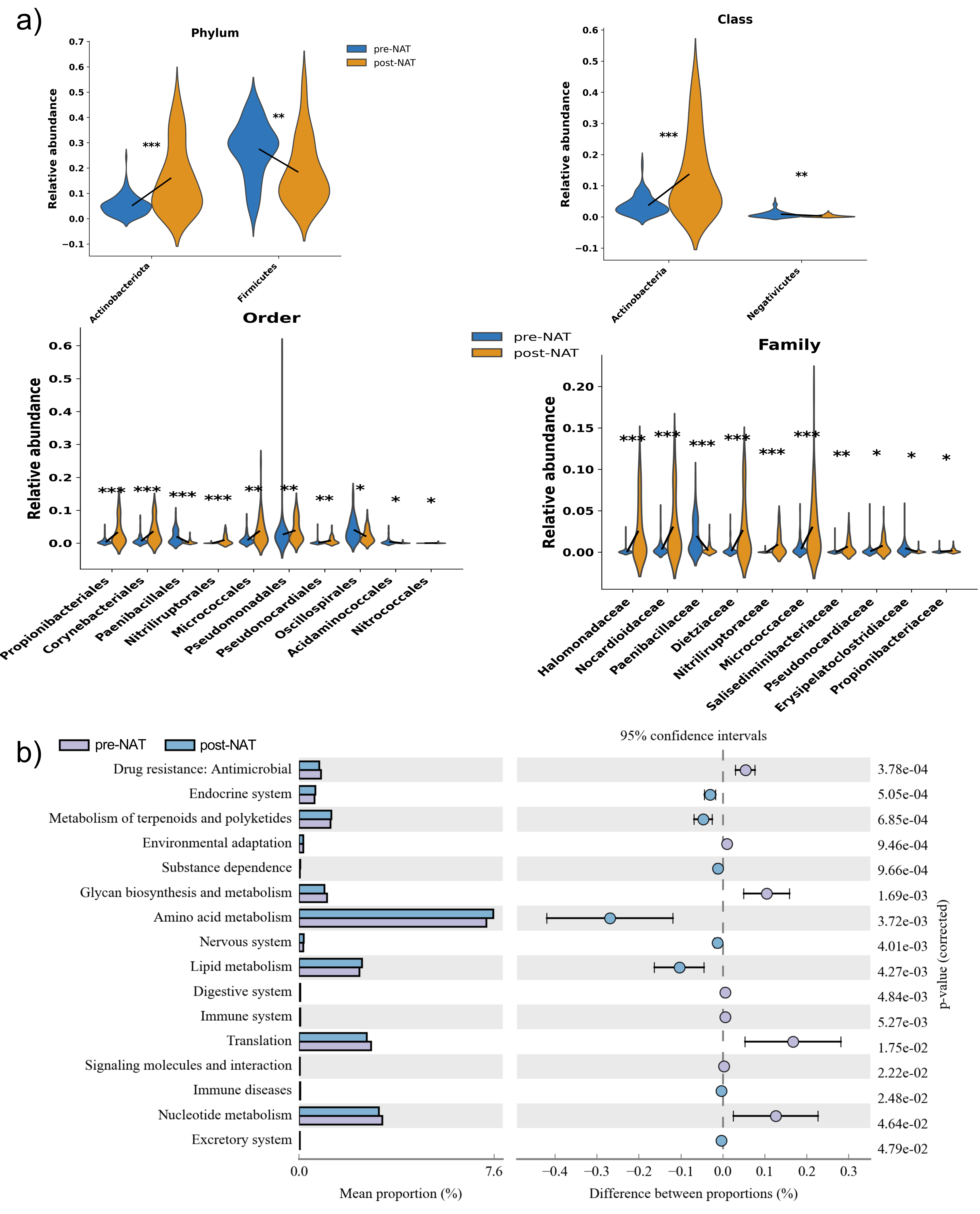

Supplement: Supplementary file 6 — Supporting Information [file CTM2-15-e70492-s007.TIF]

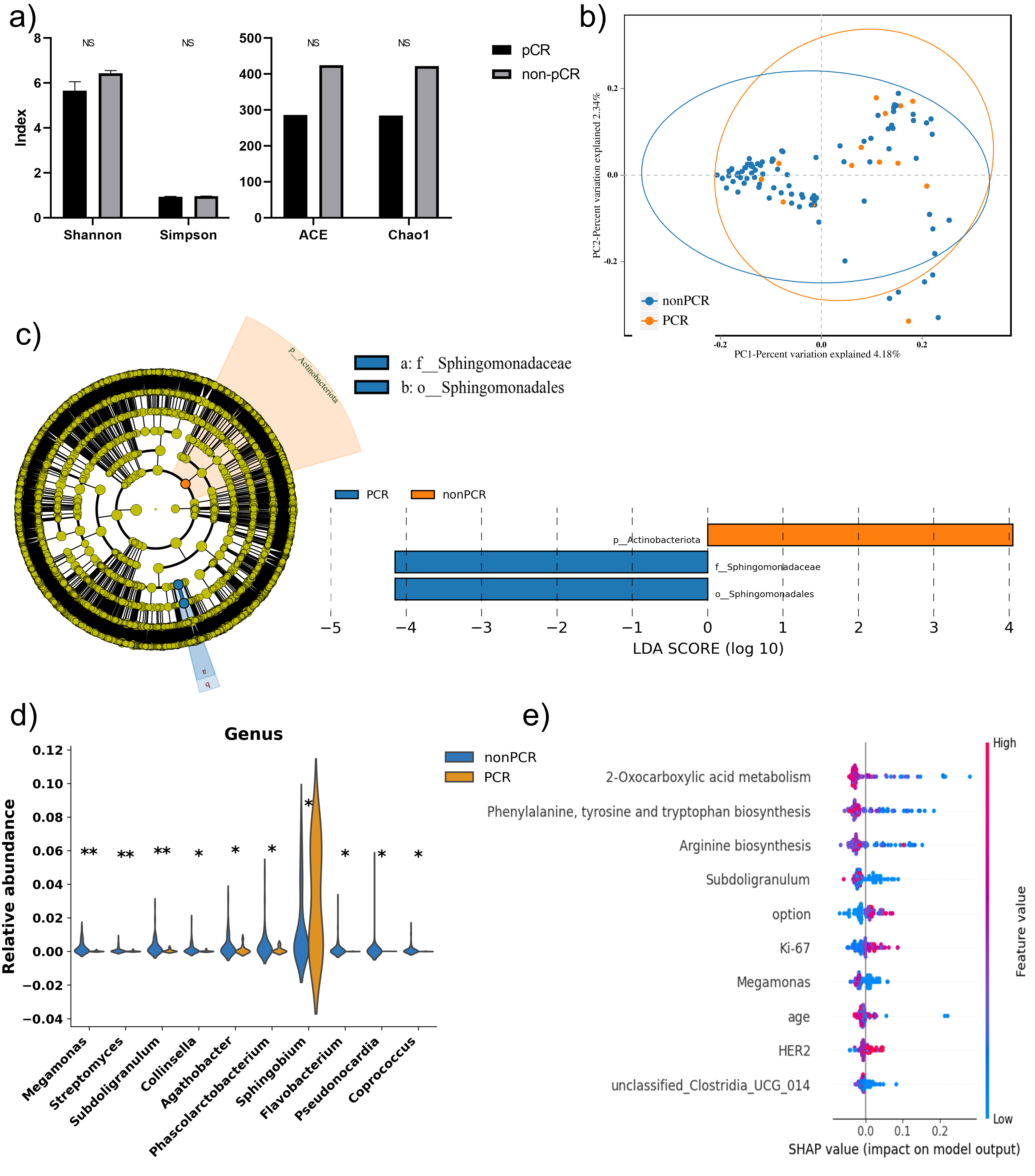

Supplement: Supplementary file 7 — Supporting Information [file CTM2-15-e70492-s006.tif]
