# Supplementary material for: Dynamics of the intratumoural microbiome across malignant transformation and treatment in breast cancer
Source: Clin Transl Med. 2025 Oct 1;15(10):e70492. doi: 10.1002/ctm2.70492 (PMC12485822; doi:10.1002/ctm2.70492)
Supplement: Supplementary file 9 — Supporting Information [file CTM2-15-e70492-s009.doc]

**Suppoting Information**

**Figure S1** Multi-dimensional comparison of tumoral microbiota between NNT and NDT groups

1a) Co-occurrence network of NNT and NDT groups

1b) Composition of microbial communities at the phylum level in NNT and NDT groups

1c) KEGG functional differences of intratumoral microbiota between NDT and NNT groups

1d)SHAP-based feature importance for predicting malignant transformation of breast nodules

Note: NNT, non-neoplastic nodule group; NDT, neoplastic development group.

**Figure S2** Intratumoral microbial differences between NDT and NNT groups

Note: NNT, non-neoplastic nodule group; NDT, neoplastic development group. * P < 0.05; ** P < 0.01, *** P < 0.001

**Figure S3** Dynamic changes of intratumoral microbiota during malignant transformation

3a) Intratumoral microbial dynamic changes from benign (NDT) to malignant (Tumor) stage

3b) KEGG functional dynamic changes from benign (NDT) to malignant (Tumor) stage

Note: NDT, neoplastic development group; T, tumor stage; * P < 0.05; ** P < 0.01, *** P < 0.001

**Figure S4** Intratumoral microbial differences between breast cancer and breast benign disease groups

* P < 0.05; ** P < 0.01, *** P < 0.001

**Figure S5** Multi-dimensional comparison of tumoral microbiota between breast cancer and breast benign disease groups

5a) Co-occurrence network of different stages

5b) Intratumoral microbial differences among different stages

Note: DCIS, ductal carcinoma in situ; * P < 0.05; ** P < 0.01, *** P < 0.001

**Figure S6** Dynamic changes of intratumoral microbiota during neoadjuvant therapy treatment

6a) Intratumoral microbial dynamic changes during neoadjuvant therapy treatment

6b) KEGG functional dynamic changes during neoadjuvant therapy treatment

* P < 0.05; ** P < 0.01, *** P < 0.001

**Figure S7** Multi-dimensional comparison of tumoral microbiota between pCR and non-pCR patients

7a) α Diversity indices between pCR and non-pCR groups

7b) β Diversity between pCR and non-pCR groups

7c) Intratumoral microbial differences identified by LEfSe analysis between pCR and non-pCR
